# Quantifying dynamic muscle lengths and moment arms of musculoskeletal joints using FEBio studio: A demonstration in the glenohumeral joint

**DOI:** 10.1016/j.jbiomech.2026.113308

**Published:** 2026-04-15

**Authors:** Breydon J. Hardy, Steve A. Maas, Brittany Percin, K.Bo Foreman, Peyton L. King, Heath B. Henninger

**Affiliations:** a Department of Orthopaedics, University of Utah, Salt Lake City, UT, United States; b Department of Biomedical Engineering, University of Utah, Salt Lake City, UT, United States; c Scientific Computing and Imaging Institute, University of Utah, Salt Lake City, UT, United States; d School of Medicine, University of California, San Diego, CA, United States; e Department of Physical Therapy and Athletic Training, Salt Lake City, UT, United States; f Department of Mechanical Engineering, University of Utah, Salt Lake City, UT, United States

**Keywords:** Muscle length, Moment arms, FEBio Studio, Shoulder, Reverse total shoulder arthroplasty

## Abstract

Muscle lengths and moment arms are necessary to quantify musculoskeletal joint torques and subsequent rotations created via muscle contractions. High-precision morphologic and kinematic data collected with dynamic stereoradiography allow semi-automatic calculation of muscle lengths and moment arms using computational methods coded into FEBio Studio. A verification/validation model of known geometry was created, simulating glenohumeral anatomy and motion, where anatomic landmarks and muscle lines of action vectors were used to calculate muscle length and moment arms dynamically throughout a motion. Moment arms were also decomposed about anatomic coordinate systems to describe the muscle’s relative contributions to physiologic planes of motion. A use case of pre- and post-operative reverse total shoulder arthroplasty was then demonstrated, where resultant changes in muscle lengths and moment arms due to the non-anatomic joint replacement agreed with prior studies. These methods, now integrated into FEBio Studio, will enable researchers and clinicians to study the effects of changing morphology and kinematics on dynamic musculoskeletal function related to muscle length and moment arms in an open-source platform.

## 1. Introduction

Muscle moment arms are the shortest perpendicular distance between a muscle line of action and joint axis of rotation, helping quantify musculoskeletal joint torques and motion and estimate muscle efficacy (Harris et al., 2022; Ingram et al., 2015; Navacchia et al., 2017). For example, the glenohumeral joint relies on dynamic interaction of eleven muscles to maintain stability during daily activities (Veeger and van der Helm, 2007). Moment arms help assess the roles of these muscles (Ackland et al., 2008). In reverse total shoulder arthroplasty (rTSA), transposing the glenohumeral ball and socket increases the deltoid’s length and moment arm to restore function in the absence of rotator cuff muscles (Boileau et al., 2005; Roche, 2022). Quantifying muscle length and moment arms may then identify patient-specific deficits, optimize muscle transfers, and prioritize rehabilitation strategies since implant selection and placement affect biomechanics (Elwell et al., 2020; Glenday et al., 2019; Zitnay et al., 2024a).

Previous computational studies relied on custom algorithms to examine muscle lengths and moment arms, typically using generic anatomy and/or static poses, omitting muscle wrapping, or reporting only moment arms (Hamilton et al., 2015; Herrmann et al., 2011; Lee et al., 2020; Levin et al., 2023; Menze et al., 2025; Navacchia et al., 2017). Subject-specific anatomy, implants, and kinematics vary, so generic data yields generic results (Lee et al., 2024; Sulkar et al., 2022, 2023). Muscle wrapping alters muscle lengths and lines of action, affecting the magnitude and direction of applied force (Garner and Pandy, 2003). Moment arms quantify the ability to create *any* joint rotation (Lee et al., 2020; Levin et al., 2023; Menze et al., 2025; Navacchia et al., 2017); decomposing them about anatomic coordinate systems illustrates their relative contributions to motion (e.g., elevation, axial rotation), although coordinate system definitions impact interpretation (Axford et al., 2025; Ingram et al., 2015).

This study presents a computational tool for estimating physiologic muscle lines, wrapping, and length in the open-source FEBio Studio. Supporting code to quantify glenohumeral moment arms evaluates a verification/validation model and patient-specific data for Rtsa.

## 2. Methods

**Fig. 1.**
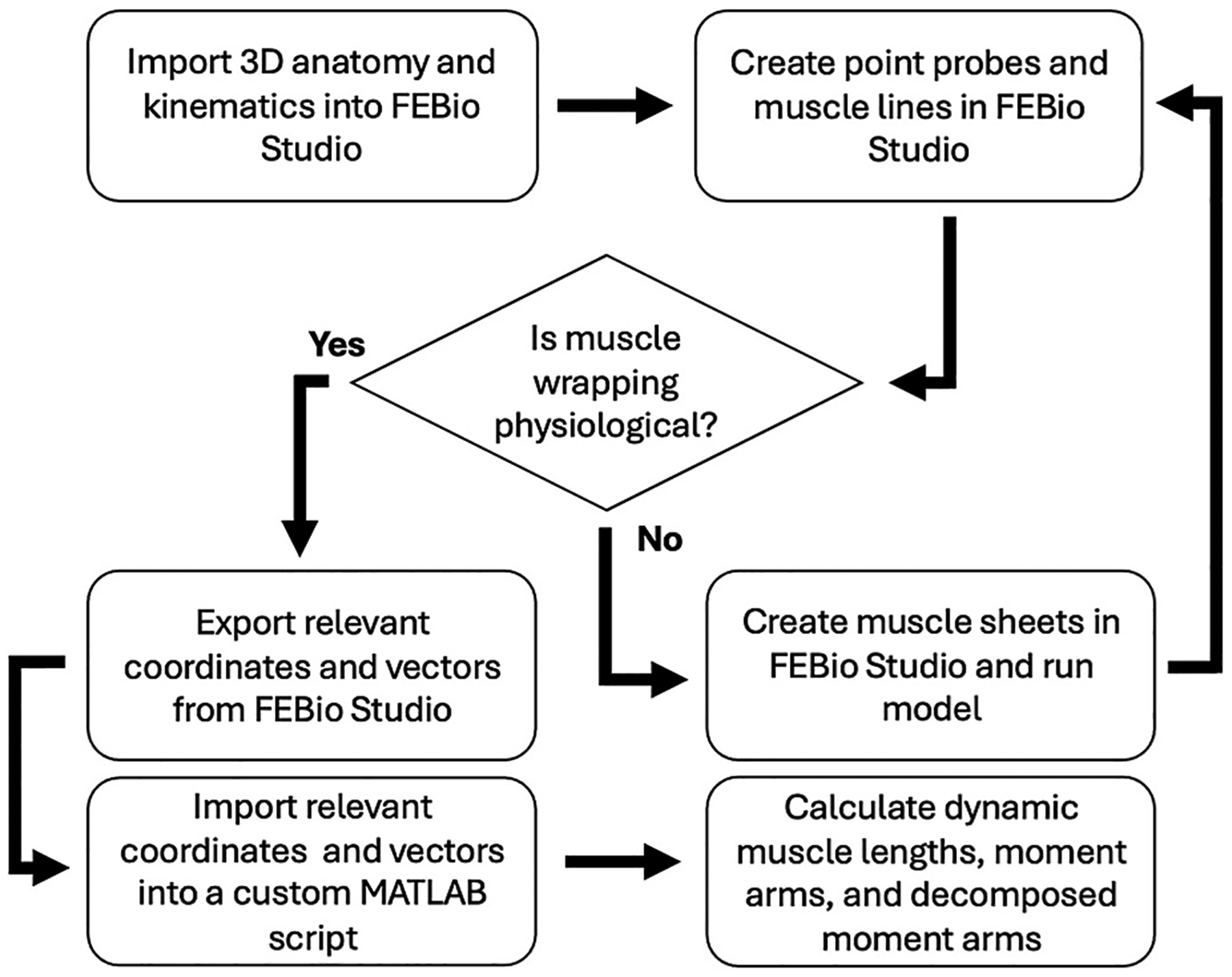
Flowchart depicting the general pipeline to move from anatomy and kinematics to a solution for their respective muscle lengths and moment arms.

**Fig. 2.**
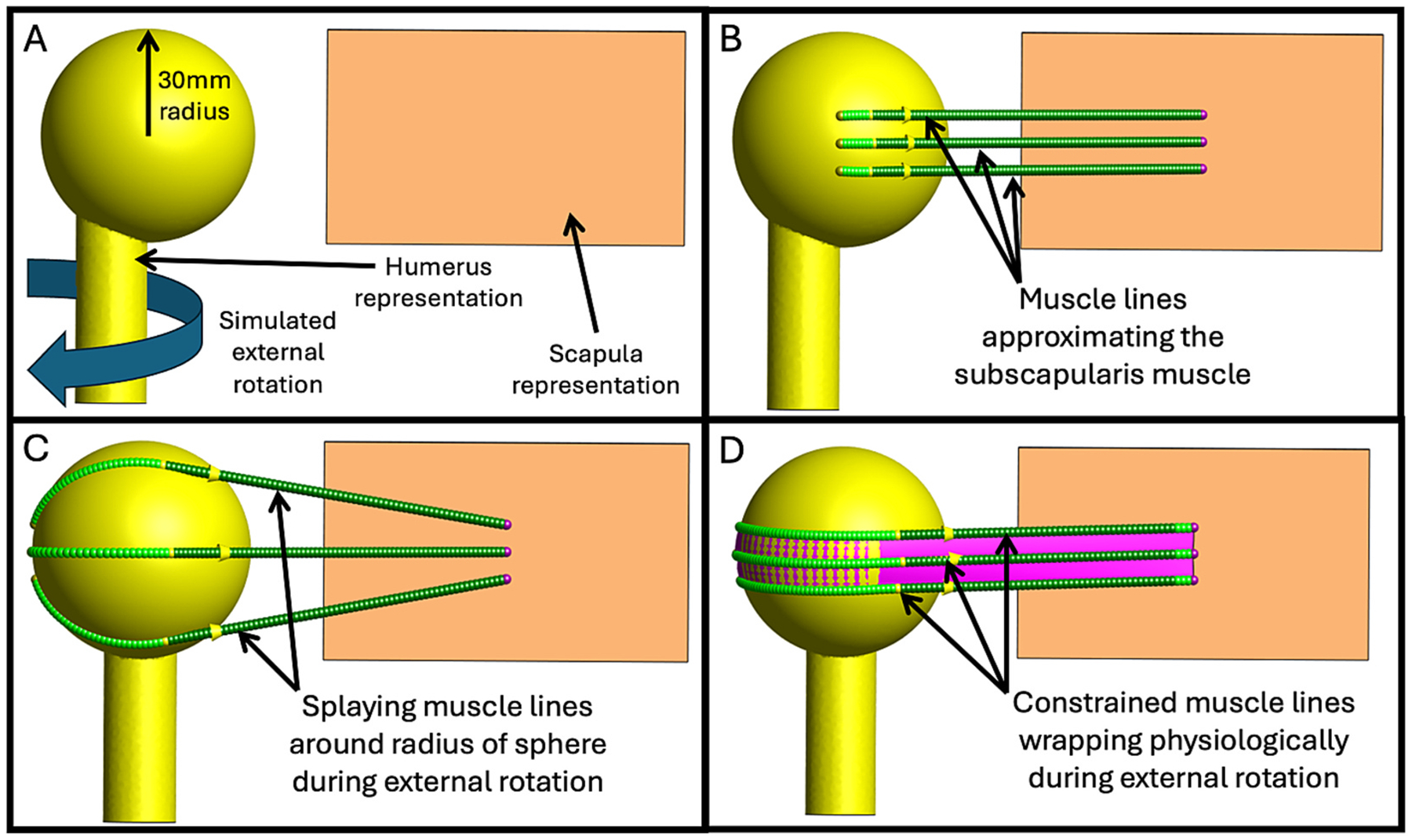
**(A)** Verification/validation model of a rectangular prism representing the scapula and a sphere with a radius of 30 mm on a stick representing the humerus, undergoing 90 degrees of simulated external rotation. **(B)** The same model at the starting position with 3 muscle lines approximating the subscapularis muscle. **(C)** The model at the end of simulated external rotation. Note the muscle lines splay around the surface of the sphere when calculating the shortest path, creating non-physiological muscle wrapping characteristics. **(D)** The model at the end of simulated external rotation, where a muscle sheet was used to constrain the paths of the muscle lines. Guiding the paths of the muscle lines results in more physiologically constrained muscle wrapping.

**Fig. 3.**
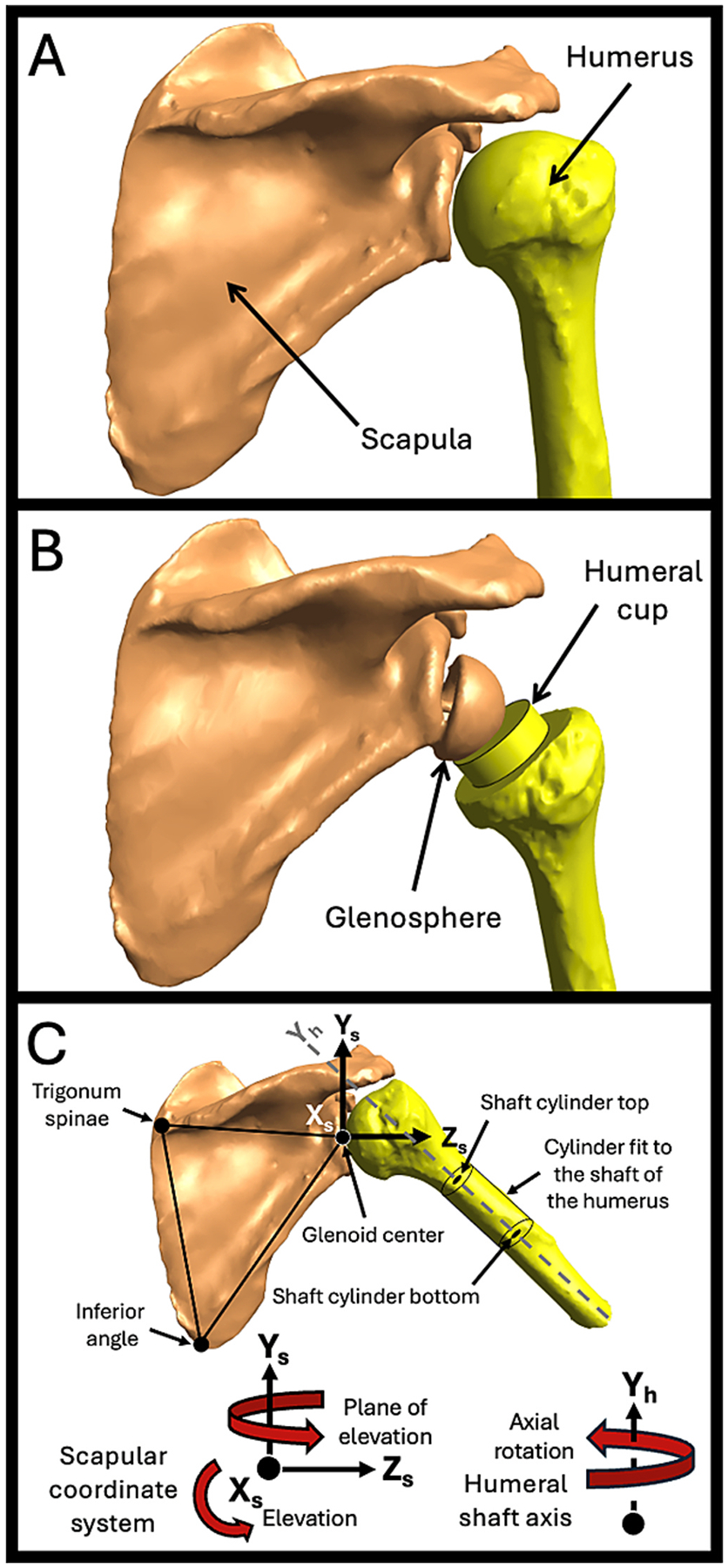
Models of the same pre-operative **(A)** and post-operative rTSA patient **(B)** at the start of scaption are displayed from the posterior view. **(C)** The trigonum spinae, inferior angle, and glenoid center (pre-op) or glenosphere center (post-op) were used to define the scapula coordinate system (Kolz et al., 2020). The scapular coordinate system followed the International Society of Biomechanics convention, except for the origin (Wu et al., 2005). The humeral head center and glenosphere center served as the center of rotation for the pre- and post-operative cases, respectively, given the fundamental functional differences of rTSA. A cylinder was fit to the shaft of the humerus to define the shaft axis since the elbow was not present, and the lesser tuberosity clocked the anterior axis (Sulkar et al., 2021). Here, rotation about X_s_ is defined as glenohumeral elevation, rotation about Y_s_ is defined as the glenohumeral plane of elevation, and rotation about Y_h_ is defined as glenohumeral axial rotation. Per the right-hand rule, elevation is negative, but the sign was reversed for ease of interpretation. All arrows denote positive rotation.

Three-dimensional (3D) bony anatomy was stored in stereo-lithography (STL) files created from segmentations of pre-operative computed tomography scans (Mimics 23.0, Materialise; Plymouth, MI, USA). The STL files were converted into LSDYNA *.k format and combined with kinematic data (*.txt) in FEBio Studio using the Kinemat tool (febio.org, (Maas et al., 2012)) (Fig. 1, Appendix: Importing Anatomic and Kinematic Data). Point probes at anatomic landmarks and muscle lines were created. Muscle line parameters were altered, and muscle sheets were introduced selectively when unconstrained muscle lines exhibited non-physiologic behavior during motion, such as snapping over bony geometry or deviating substantially from expected anatomical muscle paths. These anomalies may vary by anatomy and kinematics, requiring careful evaluation of the effects of constraint in each setting. Probe coordinates and muscle lengths and vectors were exported. A simplified model used a rectangle to mimic the scapula and a stick and sphere (30 mm radius) to simulate the humerus (Fig. 2A, Appendix: Verification/Validation Model). External rotation was applied to the sphere/stick by incrementally rotating the geometry using a rotation matrix generated via Rodrigues’ rotation formula (Fig. 2B,C). For each rotated configuration, FEBio was used to solve the muscle line configuration, both with and without sheets (Fig. 2C,D). A pre-to-post-operative rTSA patient demonstrated a clinical use case (Fig. 3A,B) (IRB #71782) (Sulkar et al., 2022), performing scapular plane abduction (i.e., scaption) for comparison to previous studies (Garner and Pandy, 2001; Graichen et al., 2001; Hamilton et al., 2015).

Muscle lines were calculated using a custom wrapping algorithm that minimized the length of a curve between end points while respecting bone geometry (informed by (Desailly et al., 2010; Gao et al., 2002), Fig. 4). Briefly, the origin and insertion were user-selected after which an initial linear muscle line was generated and discretized into a user-defined number of segments. The algorithm iteratively migrated each node towards the average of its neighbors, then projected them back onto the bone surface(s) if there was penetration. The algorithm continued until a stable configuration was found.

The subscapularis was modeled using three lines, with superior and inferior boundary lines representing the superior and inferior compartments, and one bisecting line representing the middle compartment (Hamilton et al., 2015; Herrmann et al., 2011; Netter, 2022). To quantify the moment arm, the departure point (final node contacting the humerus) was identified, as was the unit vector quantifying the direction of force along the line at that point. (Appendix: Generating Point Probes and Muscle Lines).

**Fig. 4.**
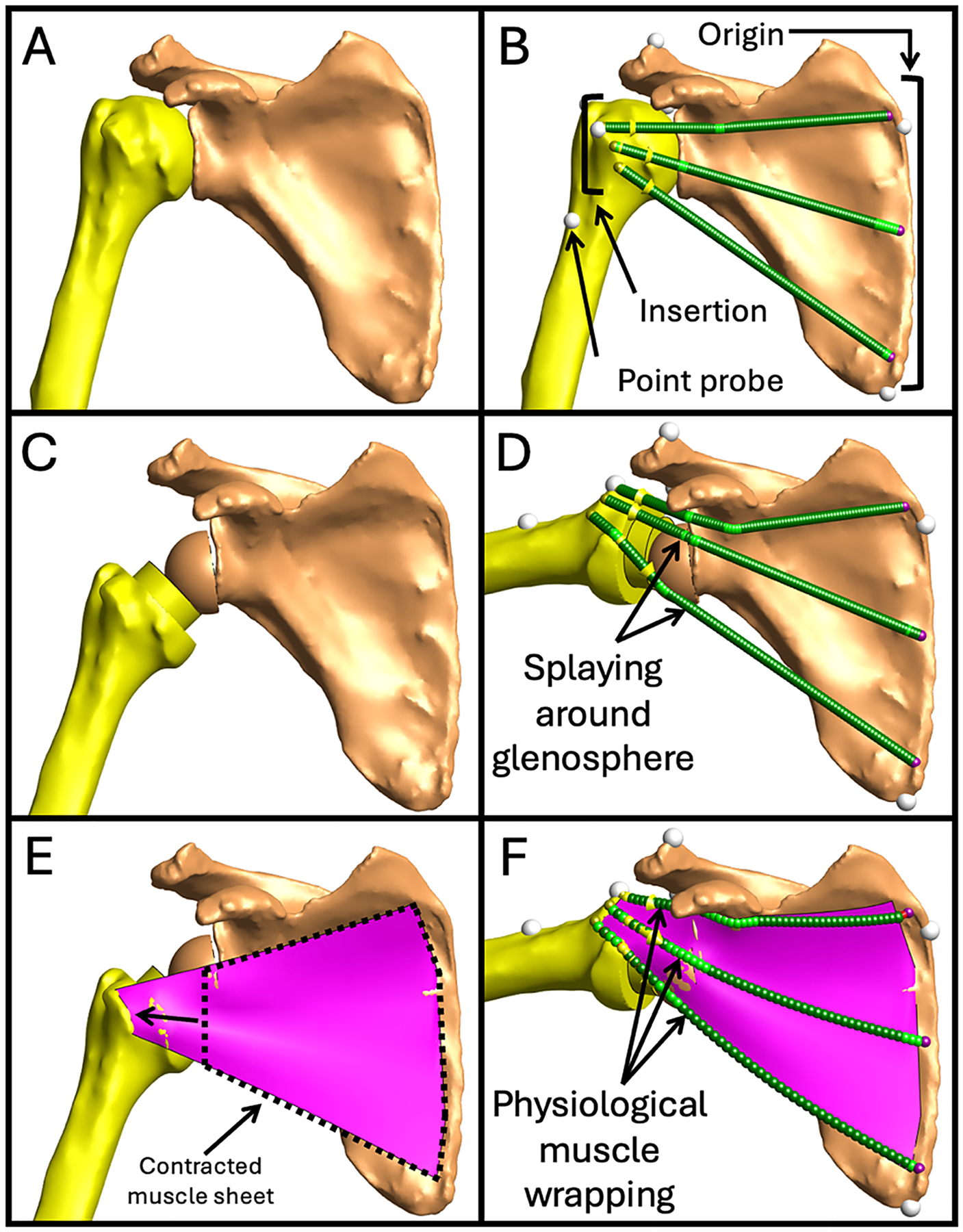
Models of muscle lines and sheets in an rTSA patient. **(A)** Patient anatomy pre-operative to rTSA. **(B)** The same model with muscle lines representing the subscapularis. Beads represent 100 individual line segments, which provided adequate muscle wrapping characteristics for unconstrained muscle lines (Appendix: Comparison of Differing Muscle Line Subdivisions). Light green beads represent where the muscle is contacting the scapula, and yellow beads represent the departure point of the muscle from the humerus (i.e., the last point of contact between the muscle line, where the moment acts). Point probes in white denote the glenoid center, trigonum spinae, inferior angle, acromioclavicular joint, lesser tuberosity, greater tuberosity, and crest of the humerus. Hidden probes also tracked the posterolateral acromion, humeral head center, shaft cylinder top, and shaft cylinder bottom. **(C)** Model of the post-operative rTSA patient. **(D)** The same model with muscle lines representing the subscapularis. Note how the muscle lines splay and wrap non-physiologically around the prosthetic components and bony anatomy during arm elevation. **(E)** The same post-operative rTSA model after the creation of a muscle sheet that was solved in FEBio with contact algorithms to constrain the physiologic paths of the muscle lines. The sheet was created using the same anatomic origin and insertion points of the subscapularis muscle as the muscle lines. The dashed black lines indicate the configuration of the contracted muscle sheet used to introduce pre-tension before returning the sheet to its original position (as shown). Pre-tensioning can reduce buckling in areas of high deformation or curvature because the sheet does not adapt its length like physiological muscle tissue. **(F)** The model after the pre-tensioned sheet was used to constrain the paths of the subscapularis muscle lines. Point probes are again shown.

If non-physiological wrapping occurred, a constraining sheet was created as a simplified representation of muscle whose deformation was calculated using FEBio and the same model data (Fig. 4D–F). To construct its initial shape, five equally spaced curves were created using the ‘geodesic curve’ tool in FEBio Studio, each with 50 subdivisions. The sheet was then created by lofting the curves together into a surface mesh using triangular elements (Appendix: Building the Constraining Sheet). A simple neo-Hookean material was applied (E = 1 MPa, υ = 0). Here, the primary objective was a compliant, deformable surface to wrap around contours and avoid numerical instabilities (e.g., negative Jacobians). If the stress–strain response of the sheet itself was of interest, more physiologic passive and active material properties would be warranted. In either case, the influence of material property selection should be weighted against the behavior of the sheet for any application of the present technique, with commensurate sensitivity studies. Although not always necessary, pre-tensioning helped prevent buckling in areas of high curvature by improving their wrapping behavior. Pre-tensioning was accomplished by initially shrinking the sheet, then pulling the edge to its target location in the initial time step of the FEBio solution. The shrunken surface was created using the ‘Mesh Morph’ tool, which contracted the sheet (Fig. 4E). Finally, a sliding elastic contact interface was defined between the sheet and bony geometry, allowing the sheet to deform and wrap around anatomical structures while preventing penetration. Additional details on contact modeling are available in an FEBio webinar and the FEBio Studio Manual (Maas, 2021; Maas et al., 2021). Once the sheet deformation was solved in FEBio, probes and muscle lines were modeled in the post-analysis as before. Muscle lines were defined on bone nodes at or just outside sheet boundaries; they do not require the same nodes. After FEBio solved the sheet deformation, the results were then loaded back into FEBio Studio. Muscle path lines were then projected onto the sheet: for each node of the muscle path, its closest point projection onto the sheet’s mesh was determined. The points, and as a result the muscle path, then followed the sheet’s motion.

Sheets sometimes buckled under large deformation and/or around complex geometry. This caused the muscle line, departure point, and vector to deviate from the intuitive path as the sheet folded back on itself. For this reason, the superior subscapularis muscle line was unconstrained in pre-operative models, while the bisector and inferior lines were constrained using a sheet. All lines were constrained in the post-operative model because buckling did not occur (Fig. 4D,F). It is worth reminding the reader that this is a first implementation and reporting of muscle lines and sheets in practice. Unbiased criteria should be developed in future studies to ensure that lines and sheets are used judiciously to describe the behavior of muscle lengths and moment arms in each application.

Muscle lengths were calculated automatically in FEBio Studio. Exported data were analyzed to calculate moment arms (Appendix: Calculating Moment Arms in MATLAB). All probe and departure point coordinates were transformed into the scapular coordinate system (Fig. 3). Departure points were recalculated to ensure the shortest perpendicular distance between their vector and the joint center of rotation. Having too few line segments could influence orthogonality. Decomposed moment arms were then calculated about the scapular coordinate system axes for glenohumeral elevation and plane of elevation (Fig. 3C). Axial rotation was defined about the humeral shaft, given its motion relative to the scapula.

Refer to the Appendix for details on protocols and Zenodo.org for analysis code/demonstration data (https://doi.org/10.5281/zenodo.17260261). The verification/validation and use case models are also provided with analysis code for demonstration. Additional open-access subject-specific shoulder data are also available: (Henninger, 2025a, b; Moissenet et al.,2025).

## 3. Results

In the validation model, non-physiologic splaying in unconstrained lines caused decreased nonlinear length changes compared to constrained lines (Fig. 2C, Δ ≤ 5.9 mm) (Appendix: Verification/Validation Model Results), but moment arms all closely followed the sphere’s radius (Δ ≤ 0.3 mm). Constrained lines and the bisector produced expected elevation, plane of elevation, and axial rotation moment arms (Δ ≤ 0.7 mm). However, elevation moment arms in unconstrained lines differed dramatically (Δ up to 17.7 mm or 227%), as did internal rotation moment arms, which decreased substantially (Δ up to 13.2 mm or 46%). Figures visualizing these results are provided in the Appendix (Appendix: Verification/Validation Model Results).

**Fig. 5.**
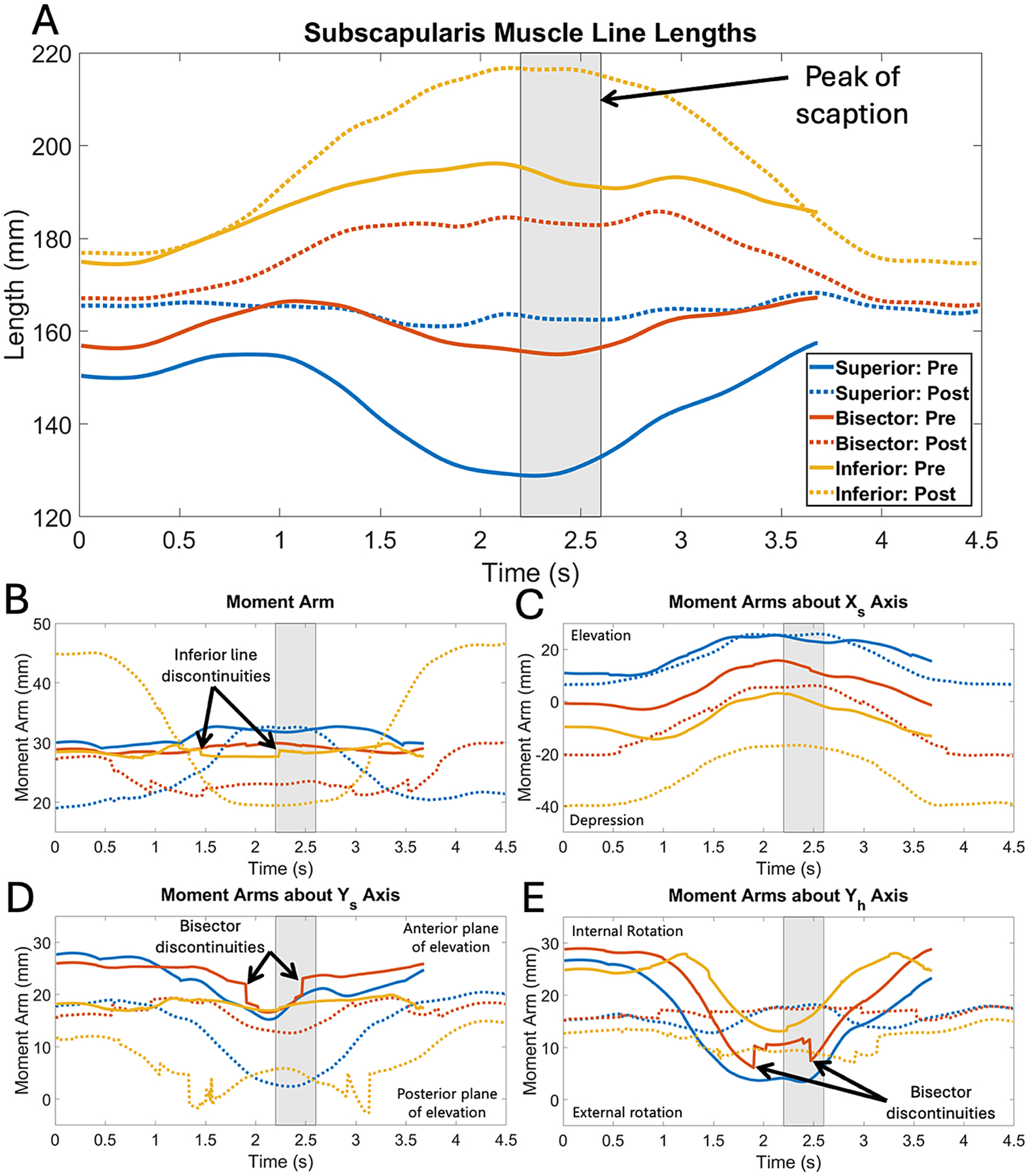
Muscle lengths **(A)**, moment arms **(B)**, and decomposed moment arms **(C-E)** for the three subscapularis muscle lines in the pre-operative and post-operative rTSA patient plotted over time in seconds. Note time is used instead of humerothoracic range of motion as is otherwise typical for the shoulder because it provided more reliable data registration for this particular subject and activity. Pre-operative subscapularis is represented as solid lines, while the post-operative is represented as dashed lines. The superior muscle lines are shown in blue, bisectors in red, and inferior lines in yellow. The peak of both pre- and post-operative scaption occurs in the shaded gray box (the peak of pre-operative scaption occurs at 2.3 s and the peak of post-operative scaption occurs at 2.5 s due to relative speed differences in performing the activities in the lab. Discontinuities in the pre-operative inferior and bisector subscapularis muscle lines are pointed out in the moment arm, moment arm about Y_S_, and moment arm about Y_h_ graphs, although they also occurred occasionally in other lines. While not typically associated with primary elevation/depression functions in the shoulder (e.g., panel C) the subscapularis does provide an antagonistic effect to the deltoid as highlighted by the post-operative shift in moment arms toward depressive function. This would be scaled by the relative muscle activation and force producing capability, which is also a function of muscle length, but quantification was beyond the scope of the present study.

When rTSA was evaluated, dramatic shifts in muscle length and moment arms occurred (Fig. 5). Post-rTSA muscle lines were longer at rest versus pre-operative (Fig. 5A). During pre-operative scaption, the superior subscapularis shortened, the inferior subscapularis elongated, and the bisector remained nearly constant. In contrast, post-operatively, the superior line maintained near constant length, but the bisector and inferior lines greatly increased. Peak changes exceeded 25%.

Pre-operative moment arms trended around 30 mm (the approximate humeral head radius) during scaption (Fig. 5B). The post-op superior subscapularis moment arm decreased by nearly 40% at rest but approximated the pre-op moment arm at peak scaption. The post-op bisector moment arm started with nominal change, but rapidly decreased by over 35% at peak scaption. Most dramatically, the post-op inferior subscapularis moment arm was 50% higher at rest but fell approximately 35% below the pre-operative moment arm at peak scaption.

When decomposed, elevation/depression moment arms were also affected by rTSA (Fig. 5C). The superior subscapularis performed effectively the same elevation function in both pre-op and post-op, whereas the post-op bisector and inferior subscapularis created progressively more depression moments in rTSA. Moment arms associated with anterior plane of elevation were all substantially decreased after rTSA, with the superior and inferior subscapularis contributing almost nothing at peak scaption (Fig. 5D). All subscapularis lines created internal rotation pre-operatively, with decreasing contributions during scaption (Fig. 5E). Post-operatively, they trended flatter with less than half the peak at rest.

Discontinuities arose in the bisector line (e.g., Fig. 5B,D,E) caused by sheet buckling or the departure point jumping between the articular margin and lesser tuberosity, altering the vector. Similar effects occurred in the inferior subscapularis solely due to departure point changes. This was pronounced in the superior subscapularis (not shown), motivating use of the unconstrained line. Many factors influence how muscle lines behave and the resultant moment arm calculation. Additional analyses, including how muscle line subdivisions, muscle line radii, bone smoothing, etc., play a role are provided in the Appendix (but are not exhaustive and representative of all possible scenarios).

## 4. Discussion

This study modeled dynamic 3D muscle wrapping in FEBio Studio to calculate muscle lengths and moment arms. These features are available in the current release (FEBio Studio 2.9).

A model with simplified geometry and prescribed kinematics verified and validated the protocol. Muscle lengths and resultant moment arms in unconstrained lines had relatively little error when compared to constrained lines, but followed non-physiologic paths. This became apparent when decomposing the moment arms about ‘anatomic’ axes, where errors ranged from 46 to 227%. This highlighted the utility of muscle sheets to constrain muscle lines, as they reduced errors back to nominal values. Mixed use of constrained and unconstrained lines had noted benefits in reducing non-physiologic wrapping in the rTSA example, but sheets were still susceptible to buckling in some instances. Users should consider expected soft behavior when creating and constraining muscle lines to optimize wrapping characteristics (Appendix: Comparison of Unconstrained Versus Constrained Muscle Lines).

The rTSA demonstrated a clinical use case where large changes in muscle lengths and moment arms as a function of implant configuration and kinematics were expected and compared to prior literature for validation. Here, the subscapularis length increased post-rTSA (Fig. 5A), which may reduce force production and/or tendon healing in cases of over-lengthening (Collin et al., 2022; Garner and Pandy, 2003; Goldfarb et al., 2025). Implant selection/placement will directly influence over-/under-lengthening, which could further impact functional range of motion (Boileau et al., 2005; Roche, 2022; Zitnay et al., 2024a). This and prior studies demonstrate the subscapularis creating depression/adduction as a deltoid antagonist after rTSA (Fig. 5C) (Giles et al., 2016; Zitnay et al., 2024b). Distalizing the humerus amplifies this effect by moving the lines of action farther below the glenosphere center of rotation, resisting elevation (Zitnay et al., 2024a). The anterior plane of elevation moment arm universally decreased after rTSA (Fig. 5D), making the shoulder more reliant on the anterior deltoid or pectoralis major. Decreased anterior cuff forces could also promote post-operative instability (Ishikawa et al., 2023; Tashjian et al., 2018). Post-operative internal rotation moment arms decreased at rest but increased at peak scaption (Fig. 5E), but decreased force production from muscle lengthening may counteract the moment arms at peak scaption. Medial and inferior center of rotation both affect subscapularis internal rotation performance (Hamilton et al., 2015; Herrmann et al., 2011). Interaction of these factors could manifest to limit internal rotation (Sulkar et al., 2023). These results are qualitatively consistent with prior cadaveric studies of subscapularis compartment moment arms during scaption, in which the superior compartment contributes to elevation/abduction, the middle compartment exhibits near-neutral behavior, and the inferior compartment contributes to depression/adduction (Ackland et al., 2008). Note that these relative functions change after rTSA owing to the non-anatomic configuration of the implanted shoulder (Zitnay et al., 2024a).

This technique has limitations. Calculating joint torques requires both moment arms and muscle force, which is not provided. Length-tension curves can estimate relative force changes (Ackland et al., 2012; Garner and Pandy, 2003; Levin et al., 2023), and forces can be derived from cadaveric simulators, computational models, or back-calculation (Ingram et al., 2015; Navacchia et al., 2019; Zitnay et al., 2024b). Muscles were approximated as lines and/or constrained on thin sheets. Muscle contraction changes their volume, as do underlying structures that were not modeled, altering both the line of action and moment arm. Muscle subregions could influence placement of constraints (Cavanaugh et al., 2024). Thus, there is a need for detailed validation against kinetics from tendon excursion and/or cadaveric studies. It is quite possible the muscle lines and sheets do not fully capture the local environment of forces acting on and within the muscle such that a single vector is a close but not complete characterization of moment arm acting on the joint. While sheet elements could visually penetrate bone (always < 0.3 mm), the node density prevented line penetration. While this study focused on an application in the subscapularis during dynamic glenohumeral motion, preliminary work (not shown) also created lines and sheets for the deltoid and other rotator cuff muscles, demonstrating broader feasibility. However, caution must be exercised as with any computational model, where individual applications are likely to present new and challenging use cases that require their own validation.

In conclusion, quantifying muscle lengths and moment arms is critical for understanding a muscle’s efficacy and role in joint function. We provide an open-source framework to assess these factors using subject-specific 3D morphology and kinematics. The demonstration cases provide a template for studying health and pathology and for evaluating how changes in anatomy, kinematics, or implant configuration influence muscle mechanics. Such analyses may inform pre-operative planning to optimize muscle function based on implant selection and placement, or how rehabilitation can be modified to target specific differences in muscle function. While patient-specific clinical application currently requires additional imaging, motion analysis, and model customization, this framework establishes a foundation for future integration of musculoskeletal modeling into clinical decision-making.

## Supplementary Material

Data_and_code.zip

README

## Appendix A. Supplementary data

Supplementary data to this article can be found online at https://doi.org/10.1016/j.jbiomech.2026.113308.

Supplementary materials contain data and code in support of the example problems described in the present manuscript. An actively maintained version of these materials can also be found at: https://doi.org/10.5281/zenodo.17260260.
