# Supplementary material for: Quantifying dynamic muscle lengths and moment arms of musculoskeletal joints using FEBio studio: A demonstration in the glenohumeral joint
Source: J Biomech. Author manuscript; Available in PMC 2026 Apr 28. (PMC13111909; doi:10.1016/j.jbiomech.2026.113308)
Supplement: Data_and_code.zip [file NIHMS2167395-supplement-Data_and_code_zip.zip › data_and_code_v1.1/FEBio_moment_arms_Appendix_260407.pdf]

# Appendix – Directions for Using Features in FEBio Studio

*Quantifying dynamic muscle lengths and moment arms of musculoskeletal joints using FEBio Studio: A demonstration in the glenohumeral joint*

See the following repository for the data and code described in this manuscript.
**Zenodo DOI:** *10.5281/zenodo.17260261*

## 1. Introduction

This document describes the process to define and analyze point probes, muscle lines of action, and constraining sheets in FEBio Studio (v2.9.1, febio.org, (Maas et al., 2012)), either with generic or patient-specific anatomy and kinematics. Data from point probes and muscle lines of action can then be exported and analyzed to calculate muscle lengths, moment arms, and decomposed moment arms.

## 2. Importing Anatomic and Kinematic Data

First, collect 3D morphologic data of the anatomies of interest (e.g., *.stl) and their respective kinematics in a common world basis (e.g., *.txt). Anatomies must be combined into a single file (LSDYNA keyword *.k), importing the anatomies in the order of the kinematic chain to be analyzed (e.g., scapula, then humerus). A kinemat file (ASCII file *.txt) is then where the kinematics of the chain are described in each row, over time. For example, for humeral motion relative to the scapula, each line would have 32 entries representing the 4x4 transformation matrix of scapular motion laid out in a 16x1 row, followed immediately by the 4x4 transformation matrix of humeral motion appended as an additional 16x1 in the same row. Subsequent rows are the subsequent time steps, in the same format. To import this data on the build side of FEBio Studio:

A. Open FEBio Studio and create a new model using the "Structural Mechanics" template.
B. Select the **Kinemat** tool in the **Tools** tab of the **Build** panel (Figure A1).
C. Enter the filename of the model file (*.k) and the kinematic file (*.txt).
D. Check *Transform initial state* if the model file bone geometries are not aligned the same as the start of the motion.
E. Then, press *Apply*.

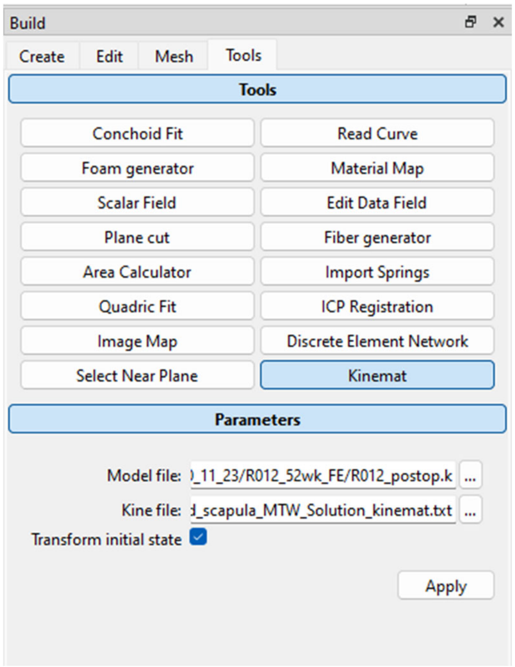


*Figure A1. Kinemat tool found in the **Tools** tab of the **Build** Panel with the model and kinematic files selected.*

This creates and assigns a rigid body to each bone. Then, kinematic data sets up rigid body displacements and rotations for all degrees of freedom. In the model panel, you will see a *rigid_displacement* or *rigid_rotation* for all six rigid body degrees of freedom. The actual data (displacement/rotation as a function of time) is stored in *loadcurves* and accessed via the Curve editor (Figure A2).

Save the model and run it to verify that the kinematics were imported correctly. To run the model:

F. Select the menu *FEBio→Run FEBio* or click the corresponding button on the main toolbar.
G. Once the model runs, FEBio Studio prompts you to open the results. Selecting *Yes* loads the plot file with the results.
    a. Plot files containing the anatomy and prescribed kinematics are generated and can be visualized to determine if the kinematics were applied correctly based on the subject-specific or generic motion.

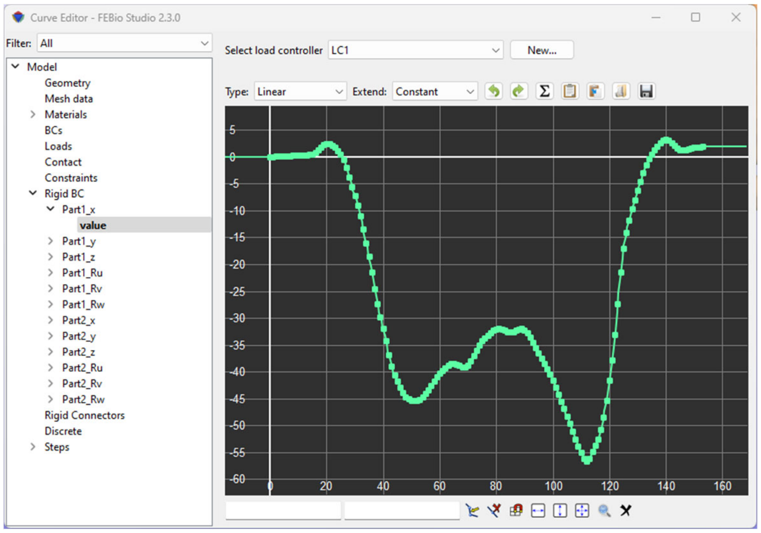

*Figure A2. Curve editor showing example load curves after the model geometry and kinematics have been imported into FEBio Studio.*

3. <u>Generating Point Probes and Muscle Lines</u>

After running the model, bone kinematics are applied, and point probes and muscle lines can be added. To add point probes and muscle lines:

A. Select *Point Probe* in the *Post* dropdown.
B. Input the coordinates for the point probe.
    a. Selecting a node, then adding a point probe, will create the point probe at the node coordinates. If no node is selected, the point probe will be created at the origin (0,0,0). Point probe coordinates can be altered by selecting the point probe in the *View* panel and inputting values for the *Initial position.* Point probes will track the nearest material by setting the *Follow* property to *Yes*.
C. To add a muscle line, select *Muscle Path* under the *Post* dropdown.
D. Input node numbers for the muscle line start and end points.
    a. Muscle lines generate without attachment points. End points are assigned to nodes by inputting the node numbers into the *Start point* and *End point* properties. *Start point* corresponds to the muscle line origin and *End point* corresponds to the muscle line insertion. The selection order of the origin and insertion is important to ensure the departure point and vector are calculated based on the insertion bone and points towards the origin.

Muscle lines were generated using a wrapping algorithm that minimized the length of a curve between two end points, while respecting bone geometry (informed by (Desailly et al., 2010; Gao et al., 2002), Figure A3). For our models, the subscapularis was modeled using three muscle lines based on its footprint, two at the boundaries and one bisector, and verified using an anatomy atlas

(Hamilton et al., 2015; Herrmann et al., 2011; Netter, 2022). Once the start and end points of the muscle line were selected, an initial estimate was created where each node migrated towards the average of its neighbors. The node was projected back onto the bone surface if it penetrated a surface. This process was repeated until a stable configuration was found.

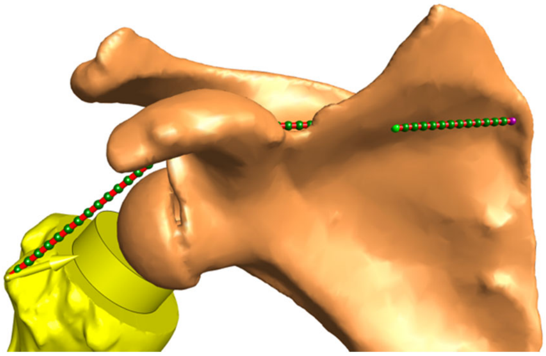

*Figure A3. Muscle line with non-physiologic muscle wrapping.*

The initial muscle line solutions may be physiologically unrealistic, i.e., wrapping around the wrong side of the bones, not following physiologic muscle paths, or penetrating bones (Figure A3). In these cases, positions of individual nodes can be manually changed, or path parameters can be altered to recalculate the muscle line path automatically.

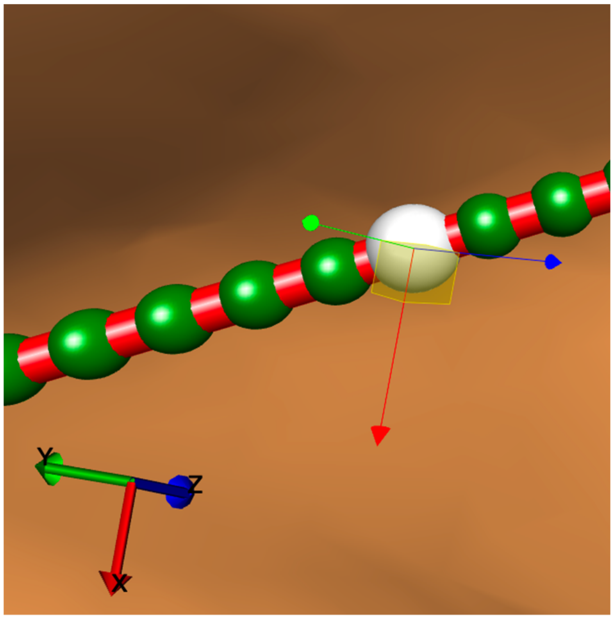


*Figure A4. The selection of a node along a muscle line corresponding to coordinate axes and planes that can be used to manually alter the position of the node.*

To manually alter muscle line node positions:

E. First, select the node.
   a. Once a node is selected, coordinate system axes will appear, and the node can be moved along those axes (Figure A4). Alternatively, a plane between two axes can be used to move the node.

F. Then, move the selected node to the desired location.
   b. Once the node is deselected, the muscle wrapping algorithm will be applied again, with the initial guess at the node's new position. Multiple nodes can be altered at once by moving nodes sequentially, without deselecting them. When all nodes have been moved, deselect the nodes, and the muscle wrapping algorithm will be applied again. This process can be repeated until the user finds an accurate muscle line path based on their use case (Figure A5).

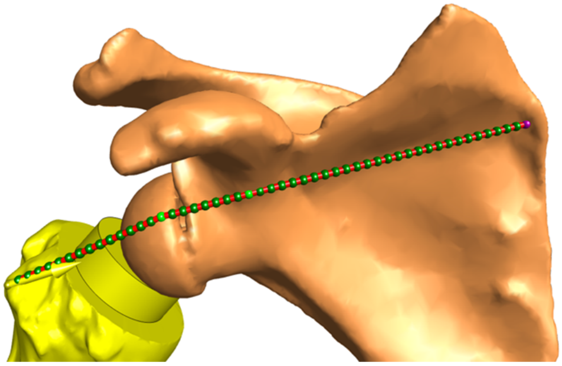

*Figure A5. Muscle line with physiological muscle wrapping after moving nodes along the muscle line.*

If non-physiological wrapping persists, path parameters can be altered to improve wrapping characteristics. To change path parameters:

G. Select a muscle line in the *View* panel.
   a. A *Properties* panel will show various modifiable graphical and computational parameters that

change wrapping and visualization characteristics.

H. Refine muscle path parameters until achieving physiological wrapping. Muscle line paths can be iteratively refined via subdivisions, maximum smoothness iterations, smoothness tolerance, snap tolerance, and search radius.

   b. Subdivisions control the number of segments per muscle line. Higher subdivisions increase the resolution of the muscle line and allow better conformity to complex surfaces and wrapping paths. More information about muscle line subdivisions can be found in *Comparison of Differing Muscle Line Subdivisions.*
   c. Maximum smoothness iterations control the maximum number of iterations used while determining a muscle line path before stopping (our models used 100). Setting maximum smoothness iterations too low may exclude more accurate wrapping paths.
   d. Smoothness tolerance controls the threshold for the smoothing algorithm and defines how small changes between iterations could be to converge (our models used 1E-06). Decreasing smoothness tolerance can allow muscle lines to converge on non-physiologic paths more easily.
   e. Snap tolerance controls the maximum distance nodes along the muscle line could snap to a surface, ensuring adherence to the bone surface without jumping too far from the previous position (our models used 1.5). Higher snap tolerances may allow muscle lines to snap to potential solutions, even if they are not physiologic.
   f. Search radius controls the radius the nodes along the muscle line search for a nearby surface. A value of 0 does not set a constraint on the search radius (our models used 0). Smaller search radii reduce how far the muscle line searches for potential wrapping paths.

4. <u>Building the Constraining Sheet</u>

Non-physiological wrapping may persist after altering node positions and path parameters, especially during dynamic motions. In these cases, we recommend using muscle sheets to constrain the muscle line paths. It is the user's responsibility to determine which method works best for their application. A comparison of unconstrained and constrained muscle lines can be found in *Comparison of Unconstrained Versus Constrained Muscle Lines.*

Our models used five equally spaced curves to build the muscle sheet framework using the same muscle wrapping algorithm described previously (Figure A6). It is up to the user to determine how many curves to use in their models. Then, the curves were lofted together, defining the sheet surface (Figure A7). Shell elements modeled the sheet, so once the surface was created, only the shell thickness needed defining.

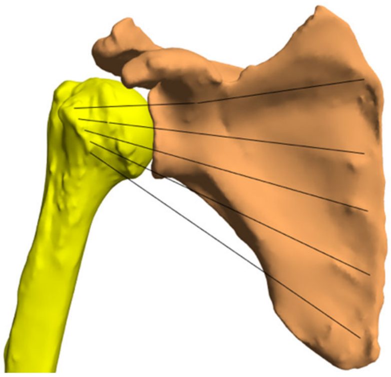

*Figure A6. Five geodesic curves that will be lofted to create the surface of the muscle sheet.*

4.1 <u>Generating muscle lines</u>

To generate curves that frame the sheet:

A. Select the *Geodesic curve* tool 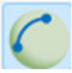 in the **Create** tab of the **Build** panel.

    a. Select the origin, then insertion on the bone mesh, and a shortest path curve will be calculated that connects the two points.
B. Then, enter the number of divisions and click *Apply*.
C. Repeat this for all desired muscle lines.
    a. The user can select as many or as few curves as they need to model the muscle of interest. Our models used five geodesic curves, each with 50 subdivisions (Figure A6).

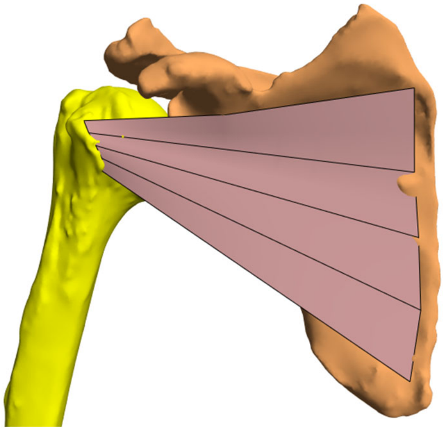

*Figure A7. The same five geodesic curves after they were lofted to create the muscle sheet.*

4.2 Creating the lofted surface

Once the curves are defined, they are lofted to create the muscle surface. To loft the curves:

A. Select the **Loft** tool 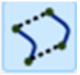 in the **Create** tab and then the curve selection tool in the top toolbar 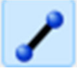.
B. Select the previously created curves.
    a. Select the curves from inferior to superior to ensure the surface's "positive" side faces the bones, which is important for the application of contact algorithms later.
C. After selecting the curves, set the element type to *Tri* and the divisions to the desired value (our model used 2), and click *Create* to loft the surface.
D. In the **Mesh** tab, click *Convert*, and select *Editable mesh* to convert the surface to a shell mesh.
    a. After creating the muscle surface, delete the original curves.
E. In the **Model** panel, find the shell part in the model tree (Geometry → Objects → LoftObject01 → Parts → and select the part corresponding to the lofted surface). Specify the shell thickness (our model used 10).

4.3 Creating the muscle material

Next, define the sheet material.

A. Select a Neo-Hookean material under *Physics* → *Add Material*.
B. Set material properties as follows: Young's modulus = 1; Poisson's ratio = 0
    a. Non-physiological values for Young's modulus and Poisson's ratio were used because the primary objective was a compliant, deformable sheet to wrap around contours.
C. To apply the material, select the surface, then assign the material by clicking the plus button in the selection pane 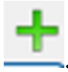.

4.4 Connecting the muscle sheet to the bones

The muscle sheet needs to be attached to the bones with rigid boundary conditions on either side.

A. Select the sheet in the graphics view, then click the node selection button 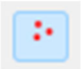 on the toolbar below the graphics view.
B. Select all the nodes on one side of the sheet corresponding to the origin or insertion.
C. Go to the menu *Physics* → *Add Nodal BC* and select **rigid**.
D. In the properties panel of the new boundary condition, set the *rb* parameter to the corresponding rigid body.
E. Clear the node selection and then repeat for the nodes on the other end of the sheet.

Now, the muscle is connected to the rigid bones. At this point, save the model and run it in FEBio again. The model does not need to run all the way to completion to verify the muscle sheet is properly connected to the rigid bones.

5. Adding the Contact Interface

A contact definition needs to be added to prevent the sheet from penetrating the bones. Contact definitions require a *primary* and *secondary* surface. The *primary* is the sheet, and the *secondary* is the rigid bone surface.

A. Go to the menu *Physics* → *Add Contact* and select *Sliding-elastic*.
B. Select the object with the bones, and then the face selection tool 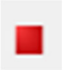.
C. Make the sheet transparent by right-clicking in the Graphics view and selecting *object transparency mode* → *unselected only*.
D. Use the face painting tool 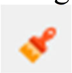 to select all the bone faces that will contact the sheet and assign them to the *secondary* (Figure A8).
E. Select all the sheet faces and assign them to the *primary*.
    a. Contact parameters can be adjusted if the contact between the muscle sheet and bones is inadequate. The parameters we altered to create physiological muscle wrapping were the *enforcement method, penalty*, *auto_penalty*, *update_penalty*, and *search_radius*.
    b. The enforcement method controls numeric contact enforcement. Our models used the Augmented Lagrangian method to improve contact interface accuracy.
    c. Penalty controls the resistance to penetration. Our models used 10 to reduce penetration. Higher penalty values can enforce contact more strictly, but may result in instability and poor convergence.
    d. Auto penalty allows FEBio to automatically set the penalty value based on material properties and mesh resolution. Our models used the auto penalty feature.

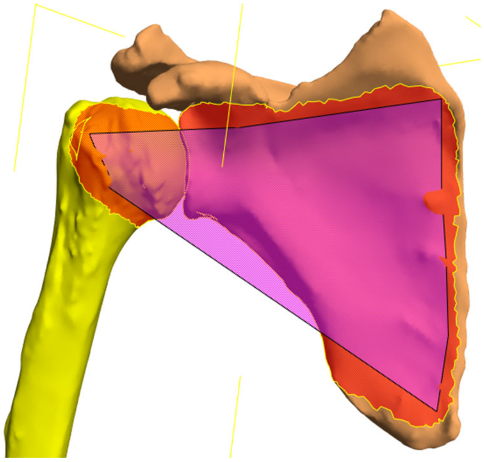

*Figure A8. Selected surfaces of the rigid bones that will be added to the secondary surface of the contact interface to ensure there is no penetration of the muscle sheet with the rigid bones.*

e. Update penalty allows FEBio to automatically update the penalty value during the simulation. Our models used the update penalty feature.
f. The search radius defines the distance the solver uses to search for contact surfaces. Higher search radii can lead to wrapping around unintended surfaces. Our models used a search radius of 2.

## 6. Muscle Pre-stressing

Buckling of the muscle sheet may occur in regions of high curvature or deformation when the sheet is not pretensioned, particularly because the sheet does not actively shorten or adapt its length in the manner of physiological muscle tissue. This behavior can alter local muscle line paths and moment arms (Figure A9). Muscle sheet pre-stressing can be implemented to prevent buckling during the loading scenario by modeling a shrunken sheet and applying a prescribed displacement during the first time step to restore the original size. If the muscle sheet only stretches throughout a motion, sheet pre-stressing is likely not necessary.

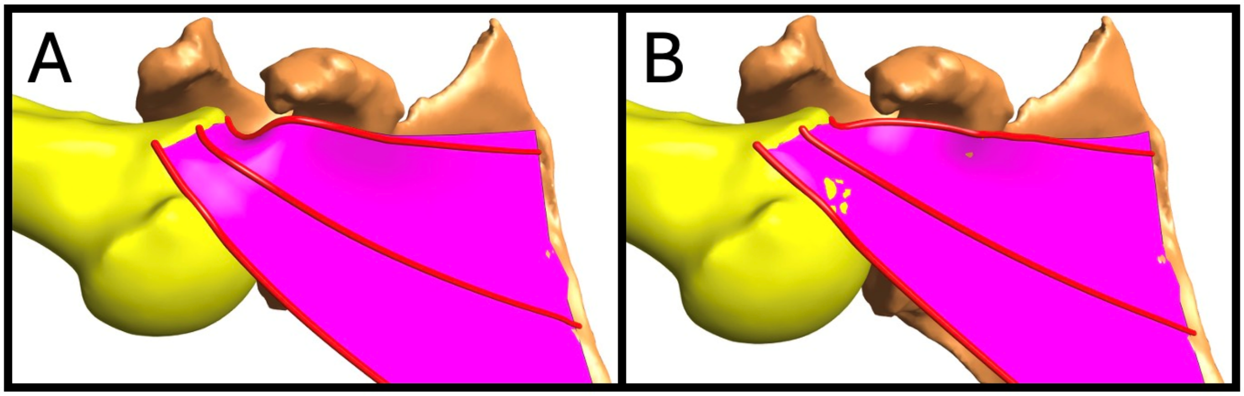


*Figure A9. (A) The pre-operative rTSA model with muscle lines constrained by a muscle sheet. The muscle sheet was not pre-tensioned and exhibits considerable buckling at the insertion. (B) The same muscle sheet was pre-tensioned and exhibits substantially less buckling at the insertions.*

### 6.1 Shrinking the muscle

A. Select the *Mesh Morph* tool on the **Tools** tab of the **Build** panel (Figure A10).
   a. This solves a small FE problem using the boundary conditions in the table. One boundary condition defines the fixed side, and another prescribes displacement to shrink the mesh.
B. Select the items on the mesh to which to apply the boundary condition.
   a. This will typically be nodes or edges for shell meshes and nodes, edges, or faces for solid meshes (our models used the edge nodes).
C. Enter the desired displacement value in the *Value* field.
   a. For instance, for fixed boundaries, enter the values (0,0,0,1). The fourth number corresponds to the weight option that locally alters the deformation by persisting the force displacement

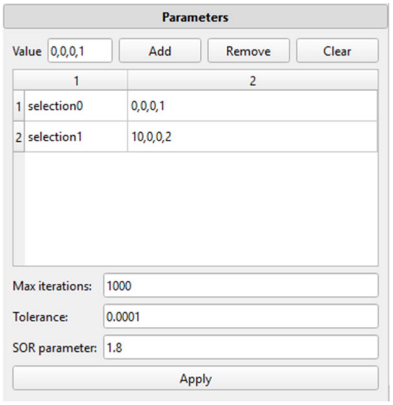


*Figure A10. Mesh Morph tool found in the* ***Tools*** *tab of the* ***Build*** *panel. Here, 'selection0' remains fixed and 'selection1' moves 10 units in the X direction. Additionally, 'selection0' has a weight of 1 and 'selection1' has a weight of 2.*

further into the mesh to reduce wrinkling during muscle sheet shrinking. Higher weights will persist the force displacement further into the mesh. Only the weight ratio affects the muscle sheet's shrinkage, i.e., 2:1 and 4:2 produce the same result. The weight should not be set to 0. Our models used 25% shrinking along the length, with a weight of 1:2. If the shrunken sheet penetrated the bones, additional displacement was prescribed until there was no penetration.

D. To add the selections, press *Add*.
   a. Edit the values by double-clicking on the value in the table. Boundary conditions can be removed by selecting the corresponding row in the table and clicking *Remove*. *Clear* removes all boundary conditions and empties the table.

E. Finally, click *Apply* to morph the mesh. If the result is not as desired, undo (menu Edit\Undo, or CTRL+Z shortcut), adjust the boundary conditions, and reapply.

6.2 <u>Updating the model</u>

To bring the sheet back to its original position:

A. Create a new analysis step (menu *Physics/Add Analysis Step*, or right-click on the *Steps* item in the model tree).

B. Select *Initial* in the field *Insert new step after*, to apply this step before the loading step.

C. Add a *zero-displacement* boundary condition on the model for any fixed boundary conditions and a *prescribed displacement* for any non-zero displacement boundary conditions applied.
   a. Note, *prescribed displacement* boundary conditions can only be applied to one degree of freedom, so multiple boundary conditions may be necessary.

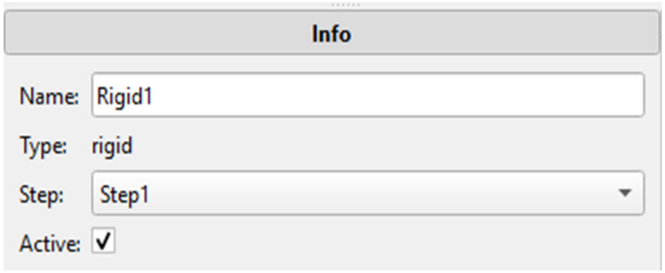


*Figure A11. Rigid boundary condition that is applied in the Initial step.*

D. Assign the contact definition to the *Initial* step so it is active during the entire analysis (Figure A11).

E. Assign the zero and prescribed displacements to the first pre-stressing step.

F. Assign the rigid interfaces to the second loading step.

G. Use the *Step Viewer* (right-click on the Steps item in the model tree) to verify that all boundary conditions are applied in the correct step (Figure A12). The Step Viewer shows a green box if the component in the left column is active during the corresponding step. Components assigned to the initial step have a green box for all steps.

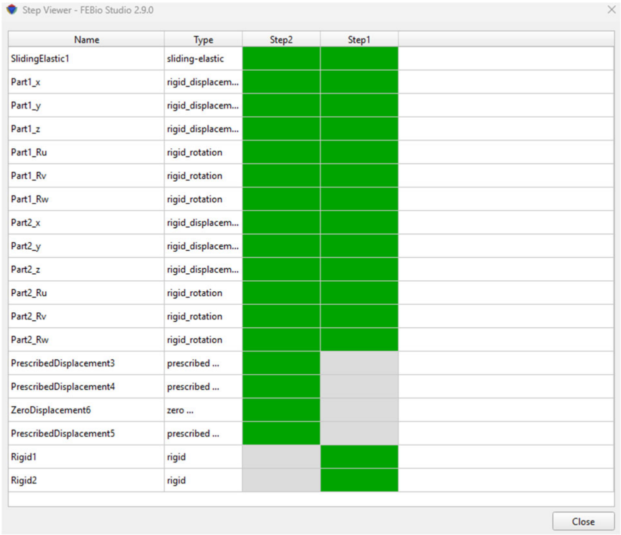


*Figure A12. Step viewer with boundary conditions applied to different steps to pre-tension the muscle sheet before the kinematics are applied.*

7. FEBio Solution Output Dependence

Before running FEBio, ensure the software only runs on one processor. When running on multiple processors, it cannot be guaranteed the order of operations will be the same. Numerical roundoff may differ between runs and affect results. Usually, the differences are small, but they may be more dramatic in ill-conditioned problems (e.g., contact). Running on one processor ensures consecutive results of the same model will be the same. All models provided and used in this paper were run using only one processor. To force FEBio to run on only one processor, set the environment variable *OMP_NUM_THREADS* to 1.

8. Running Constrained Models

To run the model:

A. Select the menu *FEBio →Run FEBio* or click the corresponding button on the main toolbar.
B. Once the model runs, FEBio Studio prompts you to open the results. Selecting *Yes* loads the plot file with the results.

If constraining sheets were used, the model may not converge or converge on a non-physiologic solution, where physiological muscle wrapping is defined as muscles following anatomically realistic paths, avoiding bone penetration, and following a smooth wrapping surface. If so, the finite element solver and contact parameters can be iteratively refined to help the model converge on a physiologic solution.

Finite element solver parameters are in the *Steps* item in the model tree. The finite element solver parameters that were altered to create the models used in this paper were line search and displacement convergence tolerance. A line search was implemented to improve convergence.

Line search parameter *ls_check_jacobians* was enabled, and checks for and attempts to resolve negative Jacobians during the line search by adjusting the time step. The line search parameter *ls_min,* the minimum line search step, must be set to 0 when *ls_check_jacobians* is enabled. Displacement convergence tolerance was increased to help the model converge (we used 0.01).

The contact parameters are located in the *Contact* item in the model tree and discussed in *Adding the Contact Interface.* Finite element solver and contact parameters may need to be iteratively refined before a stable solution with physiological muscle wrapping is found.

After obtaining physiologic results, point probes and muscle lines are added in the same way as described previously, with the only difference being guiding muscle lines with the sheet. To guide the muscle lines:

C. In the post analysis, select the muscle sheet material under the *Path guide* property.
D. Input an appropriate *Start time.*
    a. The *Start time* determines when the muscle line is constrained by the sheet, as additional timesteps may have been added if the shrunken sheet was brought back to its original position. Input a time value for when the sheet is in its original position.

Sheets may still buckle in areas of large deformation at/around complex geometry, causing deviations in the muscle line, departure point, and departure vector as the sheet folds back on itself. In these situations, it may be best to model the muscle line as unconstrained by setting the *Path guide* to *None*. This topic is further explored in *Comparison of Unconstrained Versus Constrained Muscle Lines*.

9. Saving the Model and Grouping Variables

Definitions of muscle lines and point probes are saved in a file (*.fsps) that could be applied across different activities for that subject. Muscle lines are defined at nodes on the bone surface and could be reused between activities, but point probes could only be reused if the initial bone pose was identical, as they are defined by coordinates and not node-locked.

Point probes and muscle lines can be grouped by creating a *Plot Group* from the *Post* dropdown. Creating points probes and muscle lines while selecting a plot group will add them to the plot group. Alternatively, point probes and muscle lines can be added to a plot group by right-clicking on them, selecting *Move to group,* and selecting the group to which the point probe or muscle line will be moved.

This study utilized dynamic motions; thus, position data were computed at every time step. Additionally, 'play' the model every time it is opened to store and compute the exportable data; otherwise, they will show up as '0' in the *.csv.

10. Exporting Data from FEBio

To export the point probe or muscle line data:

A. Right-click on a point probe or muscle line and select *Export data* (Figure A13).
B. A dialog box opens with an option to export the selected point probe/muscle line or all point probes/muscle lines.
C. Select the desired export option and press *Ok*.
    a. The default format for the point probe and muscle lines data is *.txt and *.csv, respectively.

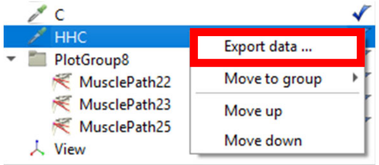

*Figure A13. Export data option for point probes.*

In the point probe *.txt file, each probe's 3D X, Y, and Z coordinates are exported at each time step, with the point probe name at the top, and separate columns for the X, Y, and Z coordinates.

In the muscle line *.csv file, muscle length and 3D coordinates of the origin, insertion, and departure point and departure unit vector are exported at each time step. Muscle length is automatically calculated by summing segments. The departure point is the final node of contact with the humerus, where the muscle force node acted. The departure vector is defined as the unit vector from the departure point to the next closest node toward the origin. The muscle line name is at the top, with separate columns for the X, Y, and Z values of each exported variable, except for muscle length, which is an integer at each time point.

11. Calculating Moment Arms in MATLAB

Exported data was analyzed by one of three included custom MATLAB scripts, one for the verification and validation model (Verification_validation_model_outputs.m), one for the pre-operative rTSA patient (*Pre_operative_reverse_outputs.m*), and another for the post-operative rTSA patient (*Post_operative_reverse_outputs.m*). The verification/validation and pre-operative script use the glenoid center to set up the scapular coordinate system and center it at the humeral head center, while the post-operative script uses the glenosphere as the center of rotation and to create the scapular coordinate system. Before running the MATLAB script:

A. Enter the exported muscle line data file name into the variable *muscle_line_file_name* and the exported point probe data file name into the variable *point_probe_file_name* in the pre- or post-operative script (Note: they must both be *.csv files).
B. Run the MATLAB script.

a. The first code section reads in point probe and muscle line data.
b. The second code section sets up the output array structure and defines point probes that will define the scapular coordinate system and humeral shaft axis. This code section also starts the loop through each muscle line and time point.
c. The third code section creates the scapular coordinate system, recasts the point probes, and defines the humeral shaft axis.
d. The fourth code section defines variables from the muscle line data and casts them into the scapular coordinate system.
e. The fifth code section calculates the moment arms and component moment arms.
f. The sixth section of the code compiles moment arms, decomposed moment arms, and muscle lengths into a final output variable that outputs as a *.csv file.

12. <u>Verification/Validation Model</u>

A verification/validation model (*Verification_Validation_model.fsps*) with well-defined geometry and prescribed kinematics ensured model and code accuracy. A rectangular prism mimicked the scapula (origins), and a stick and sphere of known (30 mm) radius simulated the humerus (insertions).

The sphere and stick were prescribed 90 degrees of pure axial rotation (about the $Y_s$ axis), away from the prism. The sphere is aligned with the $Y_s$ axis, keeping its center in the same position during rotation, simulating external rotation. Non-physiological muscle wrapping, due to muscle line splaying (Figure A14), necessitated guiding muscle lines with a constraining sheet (Figure A15).

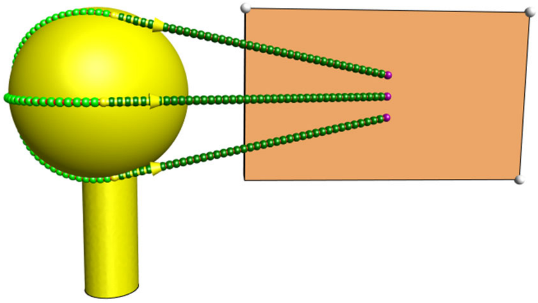

*Figure A14. Verification/validation model after external rotation was prescribed without a constraining sheet and non-physiological muscle wrapping.*

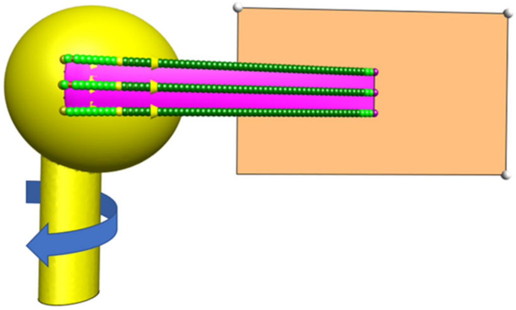

*Figure A15. Verification/validation model with a constraining sheet and muscle lines representing the subscapularis muscle. Arrow depicts external rotation of the humerus representation.*

13. Verification/Validation Model Results

Constrained muscle lines and the unconstrained bisector lengths increased linearly (0.04% error versus constrained, Figure A16). In contrast, non-physiologic unconstrained superior and inferior line splaying caused decreased, nonlinear length changes (4% error versus constrained).

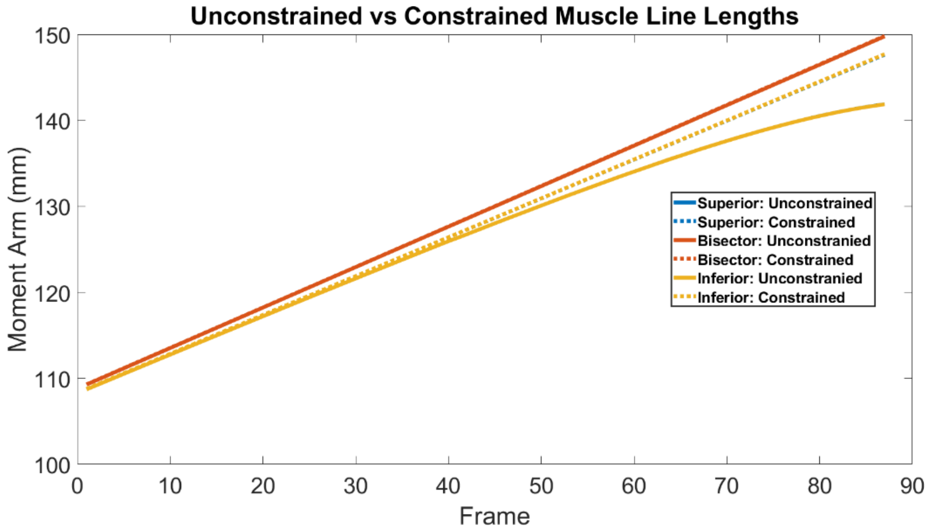


*Figure A16. Verification/Validation model comparing muscle lengths of the superior, bisecting, and inferior subscapularis muscle lines in unconstrained and constrained muscle lines during simulated external rotation, plotted over frames. The unconstrained superior (blue solid) line is directly behind the unconstrained inferior (yellow solid) line, the constrained superior (blue dashed) line is directly behind the constrained inferior (yellow dashed) line, and the constrained bisector (red dashed) is directly behind the unconstrained bisector (red solid) line.*

Both unconstrained and constrained moment arms followed the sphere's radius (error <0.03 mm, 0.1% unconstrained; <0.25 mm, 0.8% constrained, Figure A17).

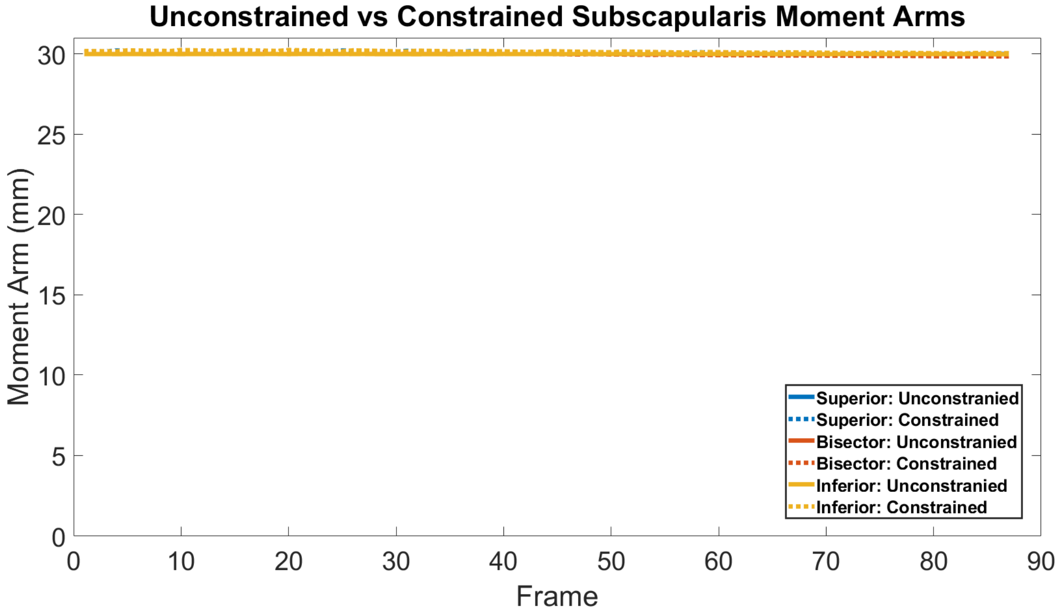


*Figure A17. Verification/Validation model comparing moment arms of the superior, bisecting, and inferior subscapularis muscle lines in unconstrained and constrained muscle lines during simulated external rotation, plotted over frames. All unconstrained and constrained subscapularis muscle line moment arms are aligned on top of each other and centered at approximately 30 mm.*

The unconstrained superior and inferior line moment arms about the $X_s$ axis were 220% greater and 227% less than constrained lines, respectively, while the constrained lines followed the expected values (<7.3% error versus expected value, Figure A18).

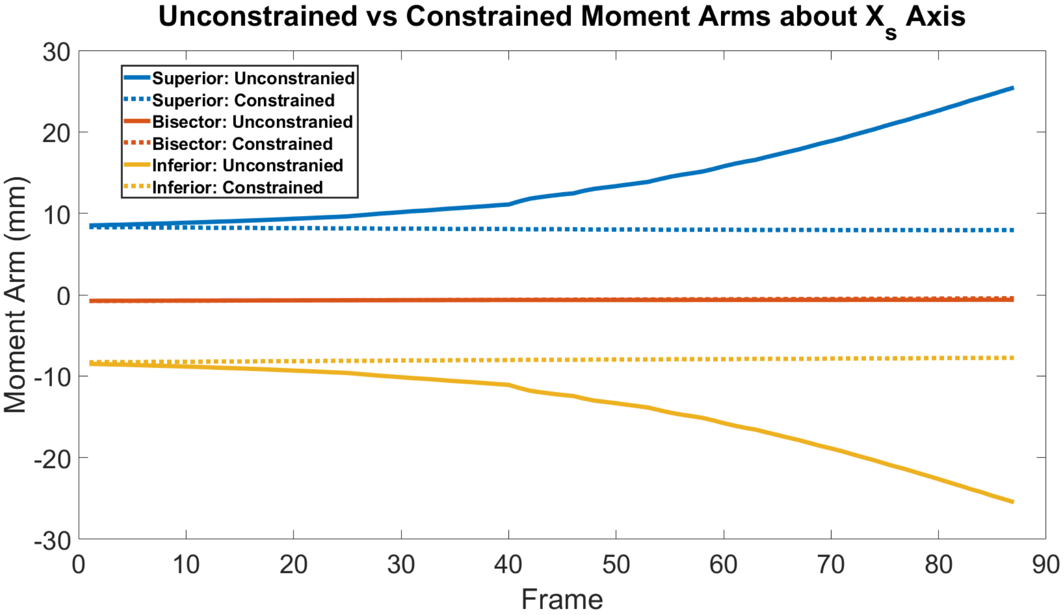


*Figure A18. Verification/Validation model comparing moment arms about the $X_s$ axis (elevation/depression) of the superior, bisecting, and inferior subscapularis muscle lines in unconstrained and constrained muscle lines during simulated external rotation, plotted over frames. The constrained bisector (red dashed) is directly behind the unconstrained bisector (red solid) line.*

Both the unconstrained superior and inferior line moment arms about $Y_s$ were 45% less than constrained, while the constrained lines followed the expected values (<1.1% error versus expected value, Figure A19).

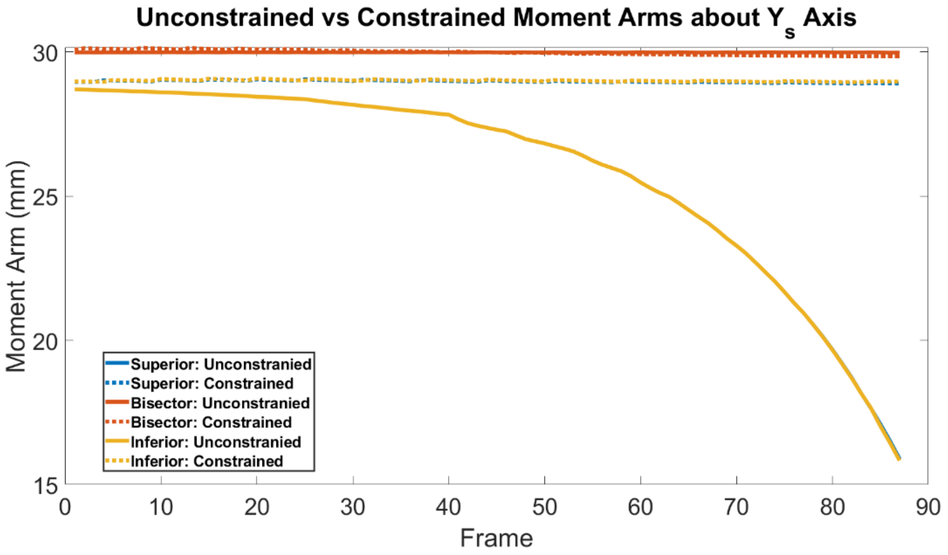


*Figure A19. Verification/Validation model comparing moment arms about the $Y_s$ axis of the superior, bisecting, and inferior subscapularis muscle lines in unconstrained and constrained muscle lines during simulated external rotation, plotted over frames. The unconstrained superior (blue solid) line is directly behind the unconstrained inferior (yellow solid) line, the constrained superior (blue dashed) line is directly behind the constrained inferior (yellow dashed) line, and the constrained bisector (red dashed) line is directly behind the unconstrained bisector (red solid) line.*

Both the unconstrained superior and inferior line moment arms about $Y_h$ were 45% less than constrained, while the constrained lines followed the expected values (<1.1% error versus expected value, Figure A20). Note: The $Y_s$ and $Y_h$ axes were aligned; thus, moment arms about $Y_s$ and $Y_h$ were identical.

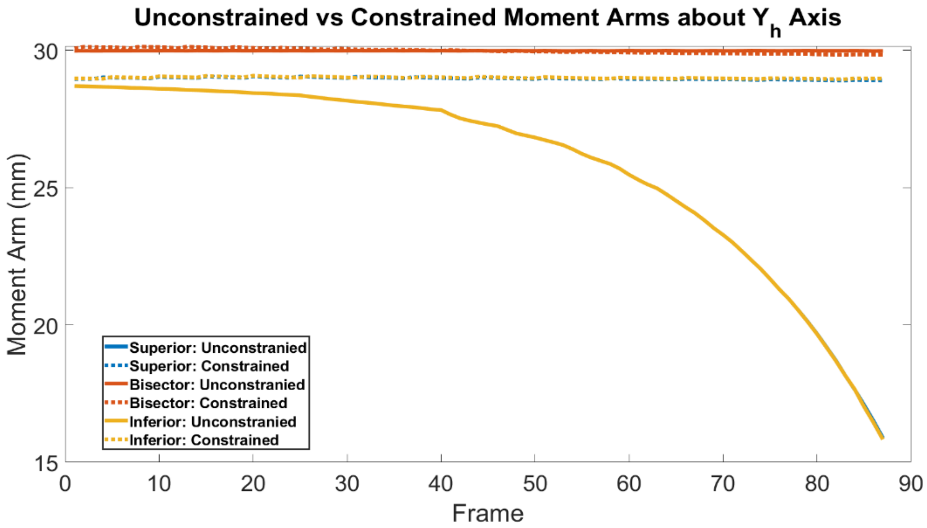


*Figure A20. Verification/Validation model comparing moment arms about the $Y_h$ axis of the superior, bisecting, and inferior subscapularis muscle lines in unconstrained and constrained muscle lines during simulated external rotation, plotted over frames. The unconstrained superior (blue solid) line is directly behind the unconstrained inferior (yellow solid) line, the constrained superior (blue dashed) line is directly behind the constrained inferior (yellow dashed) line, and the constrained bisector (red dashed) line is directly behind the unconstrained bisector (red solid) line.*

14. Comparison of Unconstrained Versus Constrained Muscle Lines

Our models presented a combination of unconstrained and constrained muscle lines to demonstrate their utility. Sheets complemented complex geometry to reduce unrealistic wrapping, but were limited in areas of high contour change or sheet rotation, creating buckling and negatively impacting departure points and vectors. Unconstrained lines succeeded when buckling occurred, if physiologic muscle paths were reasonable (e.g., Figure A21). As they can be used in combination, it is the user's job to determine which is best for their use case.

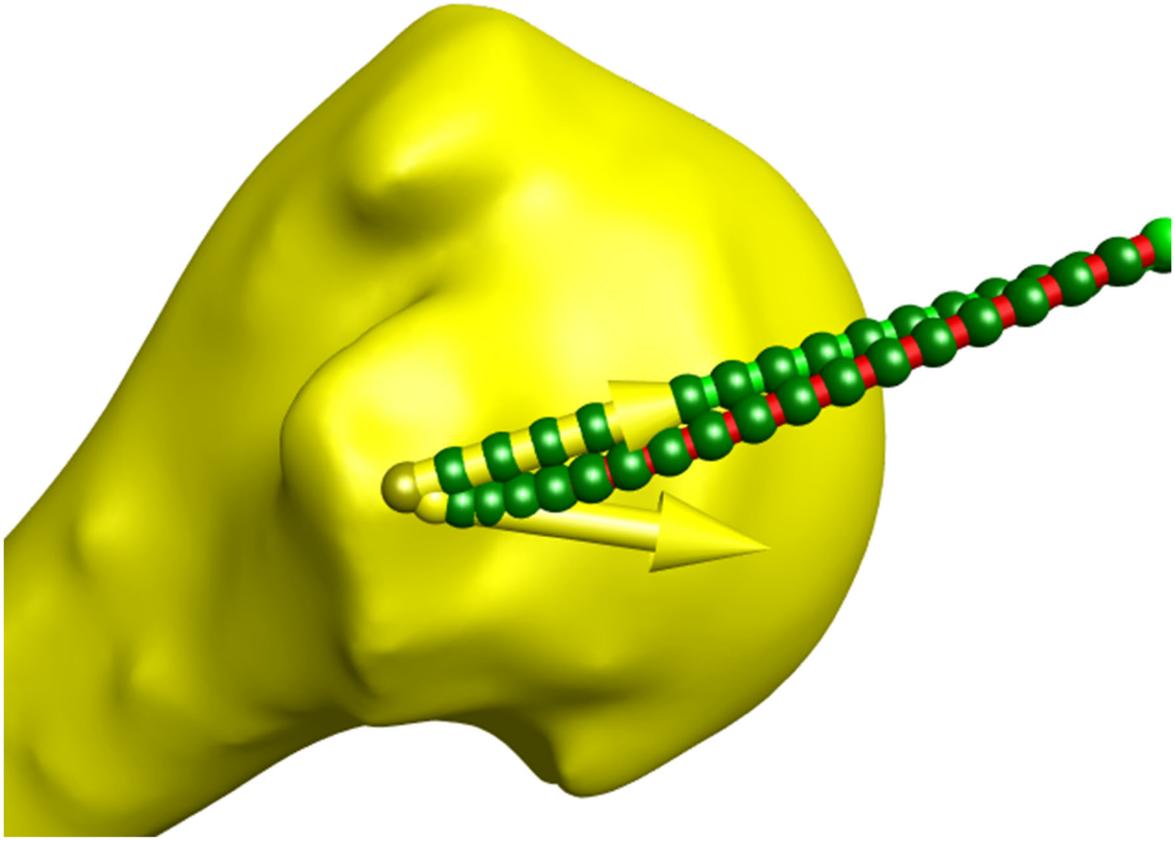

*Figure A21. Two superior subscapularis muscle lines in a pre-operative rTSA patient, one constrained (green) and one unconstrained (red). The green muscle line follows a more physiological path than the red muscle line. Note that the vectors shown highlight the deviations at the first two points of muscle insertion (not necessarily the departure point), and thus may not have large effects on the muscle length and moment arm as calculated during motion.*

Unconstrained and constrained lines were compared with the same origins and insertions to quantify differences in the modeling techniques. Results were plotted against time in seconds to visualize the complete motion and to visualize where discontinuities come from and manifest in the curves.

Unconstrained and constrained muscle lengths were very similar, differing by a maximum of 2.6% (Figure A22).

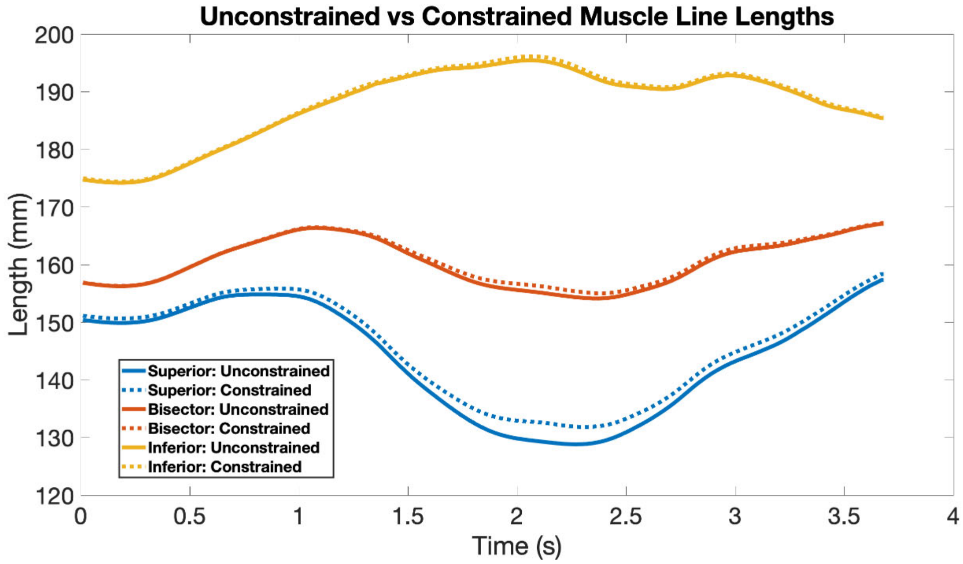


*Figure A22. Comparison of unconstrained versus constrained muscle lines in a pre-operative rTSA patient. The muscle length is plotted against time in seconds. The constrained bisector (red dashed) line is directly behind the unconstrained bisector (red solid) line, and the constrained inferior (yellow dashed) line is directly behind the unconstrained inferior (yellow solid) line.*

Unconstrained and constrained muscle lines agree when the sheet did not buckle. However, discontinuities exist in the pre-operative moment arm graphs caused by buckling at the top of the sheet and the departure point moving from the articular margin to lesser tuberosity (Figure A23).

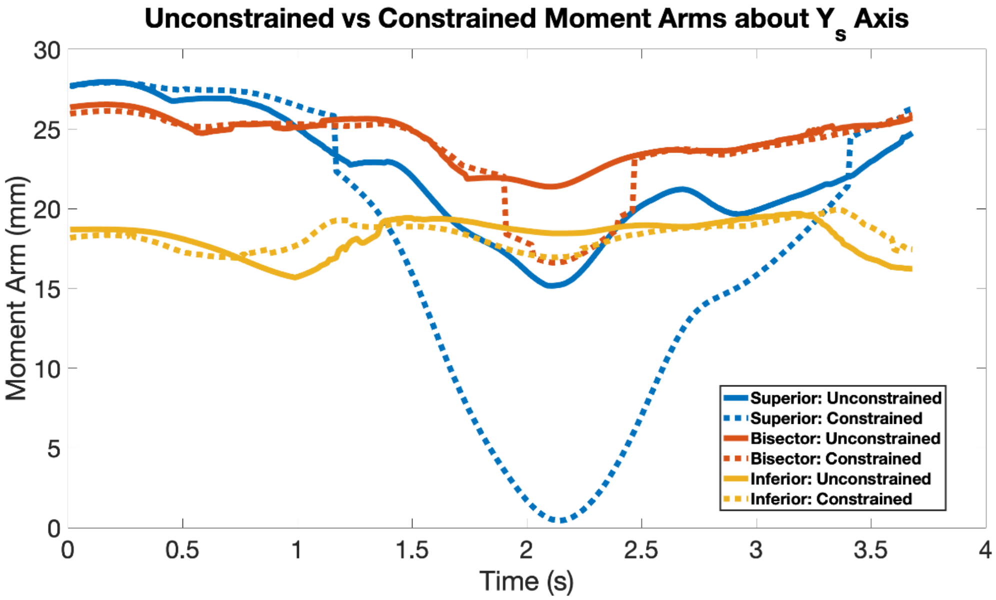


*Figure A23. Comparison of unconstrained versus constrained muscle lines in a pre-operative rTSA patient. The moment arm about the $Y_s$ axis is plotted against time in seconds. Discontinuities in the superior subscapularis muscle line occur at around 1.3 seconds, and discontinuities in the bisector occur around 2.04 seconds.*

The superior constrained subscapularis muscle line departure point moved from the 7th point along

the muscle line to the 1st point at 1.16 seconds, before moving back to the 7th point at 3.41 seconds. While at the 1st point, buckling in the muscle sheet altered the muscle line path and caused the departure vector point away from the line of action, altering the calculated moment arm and creating the discontinuity. The discontinuity in the superior line motivated the use of an unconstrained line.

A similar phenomenon occurred in the bisector, where the departure point moved between the 7th, 2nd, and 1st points, creating two sets of discontinuities.

The unconstrained superior and bisecting subscapularis muscle lines did not have discontinuities in moment arms because their departure vectors follow the muscle line of action throughout the motion, and there was no buckling.

The inferior constrained subscapularis muscle line departure point moved, but there was little to no buckling at the bottom of the muscle sheet, so the departure point followed the muscle line of action throughout motion. The constrained and unconstrained inferior lines followed similar trends.

All muscle lines were constrained in the post-operative model because sheet buckling did not considerably impact moment arms.

15. <u>Comparison of Progressive Bone Smoothing</u>

Bone models were derived from computed tomography, and the humerus was smoothed because high-fidelity segmentations interfered with contact interfaces and wrapping due to the roughness of the segmentation. The *WindowedSinc* smoothing algorithm in ShapeWorks (v6.6.1, shapeworks.sci.utah.edu (Cates et al., 2017)) was implemented to preserve bone volume and size and position of anatomical features. A *Pass Band* of 0.05 and iterations ranging from 10 to 80 were used. Smoothing amount, if any, is up to the user. Smoothed and unsmoothed humeri volumes differed <0.44% between smoothing iterations (Figure A24).

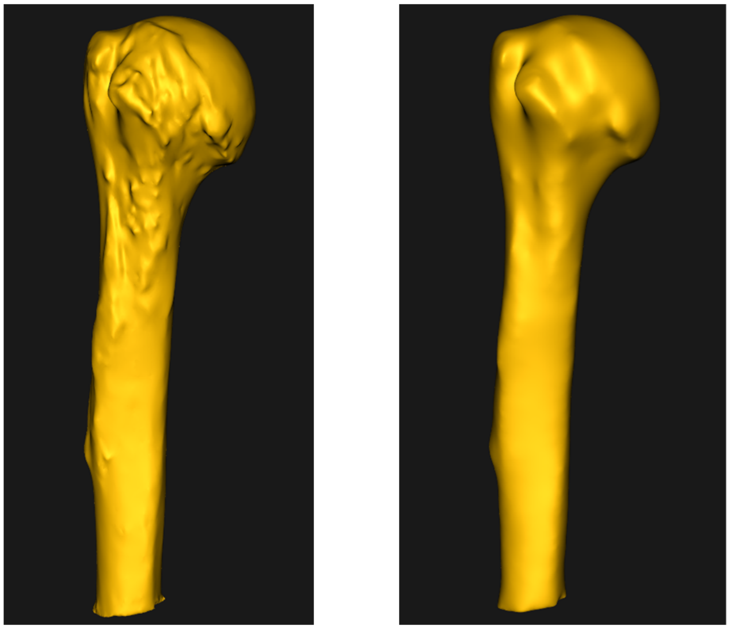

*Figure A24. Comparison of an unsmoothed humerus (left) and smoothed humerus (right). The smoothed humerus used a Pass Band of 0.05 and 80 iterations.*

Curves for all four different smoothing parameters trended similarly. Smoothed moment arm values stayed within 1.6% of unsmoothed, demonstrating its utility in assisting with solution convergence and physiological behavior while not appreciably altering the analysis (Figure A25). Our models used 80 smoothing iterations.

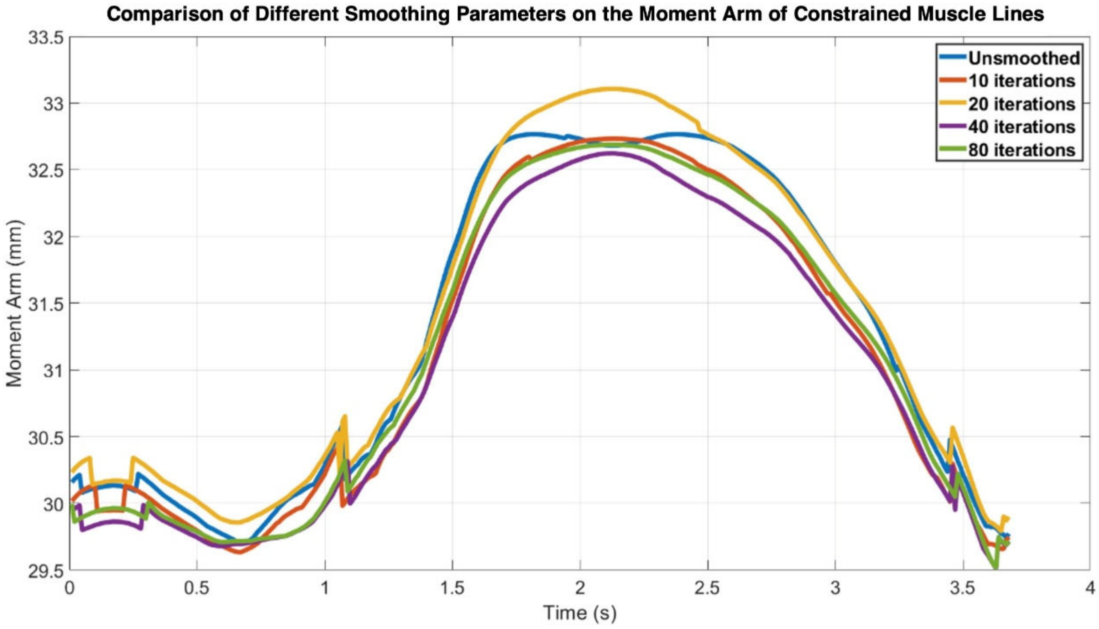


*Figure A25. Comparison of progressive bone smoothing iterations for a pre-operative rTSA patient with constrained muscle lines. The moment arm is plotted with respect to time in seconds. Smoothing iterations of 0, 10, 20, 40, and 80 are shown.*

16. <u>Comparison of Differing Muscle Line Subdivisions</u>

Muscle line subdivisions control the number of muscle line segments and can affect calculated moment arms. Different numbers of subdivisions with muscle sheet buckling created different departure vectors, due to different departure points and number of muscle line segmentations influencing the location of the next nearest node (Figure A26). Note that increasing muscle line subdivisions did not always cause the departure vector trend in the same direction because increasing segment density could change the departure point location.

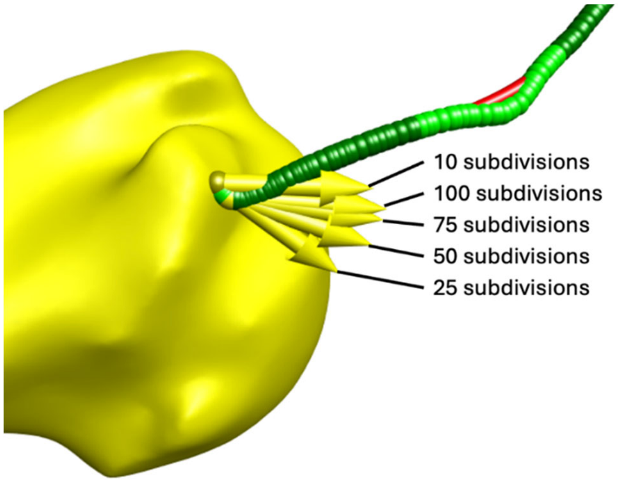


*Figure A26. Comparison of different muscle line subdivisions on the calculated departure point. Muscle line subdivisions of 10, 25, 50, 75, and 100 are shown. Note that the vectors shown highlight the large deviations at the first two points of muscle insertion (not necessarily the departure point), and thus may not have large effects on the muscle length and moment arm as calculated during motion.*

Different numbers of subdivisions changed the calculated moment arms in constrained lines (<3 mm error, Figure A27). Too few resulted in inaccurate departure points, but too many, combined with sheet buckling, create physiologically unrealistic departure vectors. Our constrained muscle lines used 50 subdivisions.

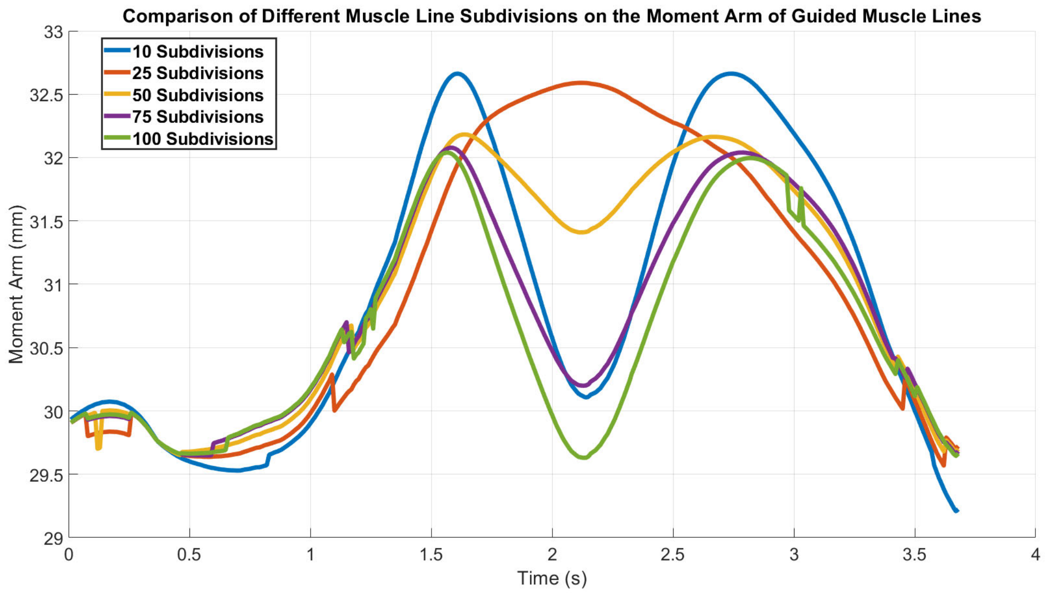


*Figure A27. Comparison of differing numbers of subdivisions in constrained muscle lines on the moment arm of the superior subscapularis muscle line in a pre-operative rTSA patient. Subdivisions of 10, 25, 50, 75, and 100 are shown.*

Different numbers of subdivisions changed the calculated moment arms in unconstrained lines (<1 mm error, Figure A28). Too few resulted in inaccurate departure points, with diminishing returns past 25 subdivisions. Our unconstrained muscle lines used 100 subdivisions.

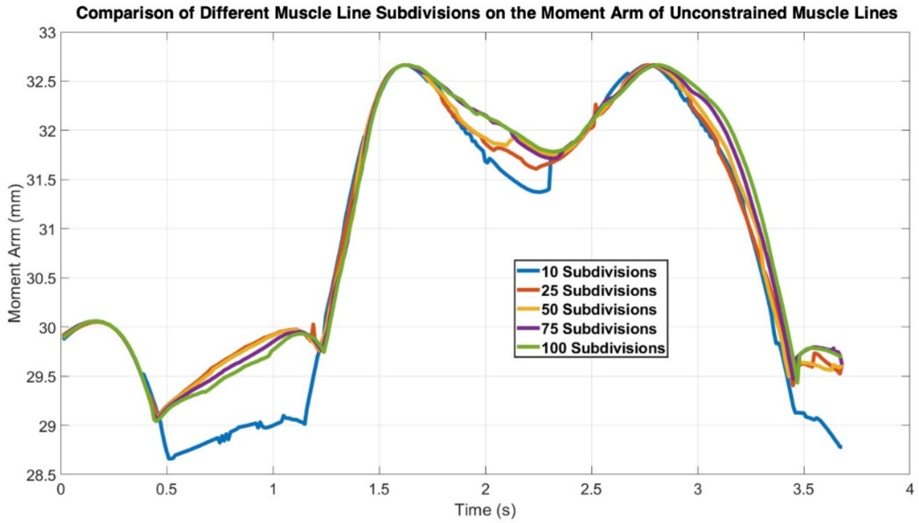


*Figure A28. Comparison of differing numbers of subdivisions in unconstrained muscle lines on the moment arm of the superior subscapularis muscle line in a pre-operative rTSA patient. Subdivisions of 10, 25, 50, 75, and 100 are shown. The 25 subdivisions (red solid) line is partially obscured by the 50 subdivisions (yellow solid) line.*

17. Comparison of Differing Muscle Line Radii

Muscle line radius controls the radius of muscle line nodes and affects constrained line moment arms. Increased line radius pushes the muscle line of action further from the bone surface and may be useful in modeling muscle thickness.

Differing radii changed the departure point, departure vector, and calculated moment arms (Figure A29). Small radii could underestimate the moment arm, while larger radii changed the time at which buckling affected the line of action. Our constrained lines used a radius of 1.

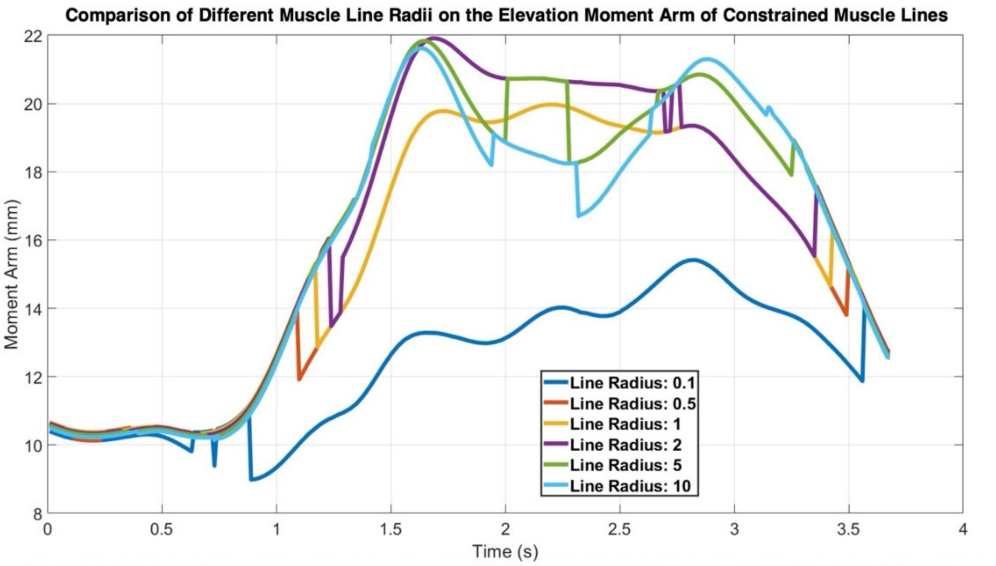


*Figure A29. Comparison of radii in constrained muscle lines on the elevation moment arm of the superior subscapularis muscle line in a pre-operative rTSA patient. Radii of 0.1, 0,5, 1, 2, 5, and 10 are shown. The 0.5 line radius (red solid) line is partially obscured by the 1 line radius (yellow solid) line.*

Differing muscle line radii did not affect the calculated moment arms of unconstrained muscle lines (Figure A30). Our unconstrained lines used a radius of 1.

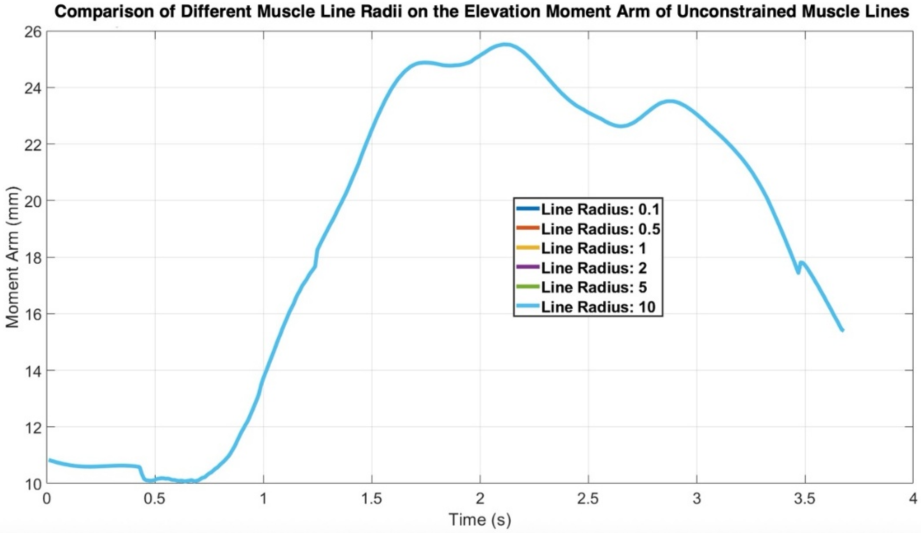


*Figure A30. Comparison of muscle line radii in unconstrained muscle lines on the elevation moment arm of the superior subscapularis muscle line in a pre-operative rTSA patient. Line radii of 0.1, 0,5, 1, 2, 5, and 10 are shown. All line radii are directly behind the 10 line radius (light blue solid) line.*

**References**

Cates, J., Elhabian, S., Whitaker, R., 2017. Chapter 10 - ShapeWorks: Particle-Based Shape Correspondence and Visualization Software, in: Zheng, G., Li, S., Székely, G. (Eds.), Statistical Shape and Deformation Analysis. Academic Press, pp. 257-298.

Desailly, E., Sardain, P., Khouri, N., Yepremian, D., Lacouture, P., 2010. The convex wrapping algorithm: a method for identifying muscle paths using the underlying bone mesh. J. Biomech. 43, 2601-2607.

Gao, F., Damsgaard, M., Rasmussen, J., Christensen, S.T., 2002. Computational method for muscle-path representation in musculoskeletal models. Biol. Cybern. 87, 199-210.

Hamilton, M.A., Diep, P., Roche, C., Flurin, P.H., Wright, T.W., Zuckerman, J.D., Routman, H., 2015. Effect of reverse shoulder design philosophy on muscle moment arms. J. Orthop. Res. 33, 605-613.

Herrmann, S., Konig, C., Heller, M., Perka, C., Greiner, S., 2011. Reverse shoulder arthroplasty leads to significant biomechanical changes in the remaining rotator cuff. J. Orthop. Surg. Res. 6, 42.

Maas, S.A., Ellis, B.J., Ateshian, G.A., Weiss, J.A., 2012. FEBio: finite elements for biomechanics. J. Biomech. Eng. 134, 011005.

Netter, F.H., 2022. Netter Atlas of Human Anatomy: Classic Regional Approach, 8th Edition ed. Elsevier.
