# Supplementary material for: Quantifying dynamic muscle lengths and moment arms of musculoskeletal joints using FEBio studio: A demonstration in the glenohumeral joint
Source: J Biomech. Author manuscript; Available in PMC 2026 Apr 28. (PMC13111909; doi:10.1016/j.jbiomech.2026.113308)
Supplement: README [file NIHMS2167395-supplement-README.docx]

**Article Title:** *Quantifying dynamic muscle lengths and moment arms of musculoskeletal joints using FEBio Studio: A demonstration in the glenohumeral joint*

**Citation:** *Accepted, Journal of Biomechanics, 15 April 2026*

**J Biomech DOI:** [*https://doi.org/10.1016/j.jbiomech.2026.113308*](https://doi.org/10.1016/j.jbiomech.2026.113308)

**Zenodo DOI:** *10.5281/zenodo.17260261*

**Henninger Lab, Harold K. Dunn Orthopaedic Research Laboratory**

**University of Utah, Salt Lake City, UT**

[**https://medicine.utah.edu/orthopaedics/research/labs/harold-dunn/groups/henninger**](https://medicine.utah.edu/orthopaedics/research/labs/harold-dunn/groups/henninger)

This repository contains data and code for analyzing muscle lengths, moment arms, and decomposed moment arms of the human shoulder, utilizing subject-specific anatomic and kinematic data. The features have been integrated into FEBio Studio (version 2.9.1, febio.org), and supporting MATLAB (version 2024a) code analyses moment arms specific to the glenohumeral joint of the shoulder as a demonstration case.

These techniques can be applied to data found in the following repositories that contain high precision anatomic surfaces and kinematics of the shoulder:

- healthy shoulders ([***https://doi.org/10.5281/zenodo.14889478***](https://doi.org/10.5281/zenodo.14889478))
- shoulders after rTSA ([***https://doi.org/10.5281/zenodo.16537557***](https://doi.org/10.5281/zenodo.16537557))

**File Naming Conventions**

1. ‘Verification_Validation’
   1. Well-defined geometry and prescribed kinematics to ensure the accuracy of calculated muscle lengths and moment arms.
2. ‘Pre-operative_reverse’
   1. De-identified data from a patient prior to receiving a reverse total shoulder arthroplasty (rTSA).
3. ‘Post-operative_reverse’
   1. De-identified data from the same patient 52 weeks after receiving an rTSA. The patient was assumed to be functionally recovered by 52 weeks.

**Files Types**

1. Anatomic models (found in the folder ‘model_source_files’)
   1. Morphologic data for all moving bodies combined in a single file to be read by FEBio Studio (LSDYNA Keyword *.k).
   2. Source surfaces (*.stl) and pre-generated anatomic models (*.k) can also be found in the Zenodo repositories referenced above.
   3. Follow the steps detailed in the manuscript Appendix to generate a complete FEBio Studio model (*.fs2) using these files.
2. Kinematics (found in the folder ‘model_source_files’)
   1. Kinematic data for all moving bodies combined in a single file to be read by FEBio Studio (ASCII file *.txt). Note that the ordering of kinematic data in this file must be consistent with the ordering of moving bodies input to the *.k file.
   2. Pre-generated kinemat files (*.txt) can also be found in the Zenodo repositories referenced above.
   3. Follow the steps detailed in the manuscript Appendix to generate a complete FEBio Studio model *.fs2) using these files.
3. FEBio Models and Output Files
   1. FEBio Studio Files (*.fs2) – the executable FEBio studio model file format.
   2. FEBio Input Files (*.feb) – the FEBio input file format for solving muscle sheet contact problems.
   3. FEBio Plot Files (*.xplt) – the FEBio output format used to store results.
   4. FEBio Post Session Files (*.fsps) – the post-session file that stores the results and all other items added (i.e., point probes and muscle lines).
4. ‘Jobs’ folder
   1. Created each time an *.fs2 file is run. Contains the *.feb, *.xplt, and *.log files generated from executing the *.fs2 file.
5. Analysis Code Inputs (i.e., outputs from FEBio Studio)
   1. Point Probe Output Data (*_probes.csv)
      1. A file containing each point probe’s 3D XYZ coordinates for each time point in a model trajectory.
      2. This file is generated from within the *.fsps file. Follow the steps detailed in the manuscript Appendix to generate this file.
   2. Muscle Line Output Data (*_muscles.csv)
      1. A file containing each muscle line’s length, origin, insertion, and departure point 3D XYZ coordinates, along with the departure unit vector for each time point in a model trajectory.
      2. This file is generated from within the *.fsps file. Follow the steps detailed in the manuscript Appendix to generate this file.
6. MATLAB Analysis Code (i.e., takes the *_probe.csv and *_muscles.csv files as input)
   1. Verification_validation_outputs.m
      1. MATLAB code used to calculate moment arms and decomposed moment arms for the verification and validation model. Three landmarks on the plane (i.e., virtual glenoid center, the trigonum spinae, and inferior angle) create the plane (i.e., scapular) coordinate system with the center at the humeral head (sphere) center.
   2. Pre_operative_reverse_outputs.m
      1. MATLAB code used to calculate moment arms and decomposed moment arms for pre-operative rTSA patients. The glenoid center, the trigonum spinae, and the inferior angle create the scapular coordinate system with the center at the humeral head center.
   3. Post_operative_reverse_outputs.m
      1. MATLAB code used to calculate moment arms and decomposed moment arms for post-operative rTSA patients. The glenosphere center, the trigonum spinae, and the inferior angle create the scapular coordinate system, with the center at the glenosphere center.
7. Analysis Code Outputs
   1. Output files (*.csv) from the provided MATLAB script using the provided FEBio models. Contains the muscle lengths, moment arms, and decomposed moment arms for each of the three modeled subscapularis muscle lines.
   2. Examples from the manuscript in the folder ‘provided_solutions_Matlab’) and provided for comparison to your results when running the code.

**Running the provided models in FEBio Studio**

1. Users can create *.fs2 models using the protocol provided in the manuscript Appendix, along with *.k and *.txt files from the referenced repositories. Otherwise, to execute the pre-built model from the manuscript follow these steps:
2. Download the latest version of FEBio Studio
3. Required model files from this repository:
   1. Verification_validation_model.fs2
   2. Pre_operative_reverse_model.fs2
   3. Post_operative_reverse_model.fs2
      1. Note: Running this code will generate new *.feb, *.log, and *.xplt files in the ‘jobs’ folder. For comparison, these files were previously generated and are provided in the folder ‘provided_solutions_FEBio’. If using the provided solutions, jump to step 5.
4. Open a model (*.fs2) file in FEBio.
5. Before running the *.fs2 files, ensure the environment variable OMP_NUM_THREADS is set to 1 to force FEBio to run on only one processor to ensure the results of the model will be the same (See Appendix Section 7 for more detail).
6. Then, select the menu FEBio *🡪* Run FEBio or click the corresponding button on the main toolbar (red FEBio cube icon) to run each model file.
7. Once the models run, FEBio Studio generates a prompt to open the results. Select *Open Results* to load the plot file (*.xplt) with the results.
8. In the plot file, point probes and muscle lines can be created.
   1. Although point probes and muscle lines can be created, they are not necessary because they are already included in the *.fsps file.
   2. Saving the plot file as an *.fsps file will store point probes and muscle lines so that anytime the post-session file is opened, the point probes and muscle lines will already be generated.

**Exporting point probe and muscle line data from FEBio Studio**

1. Required model files from this repository:
   1. Verification_validation_model.fsps
   2. Verification_validation_model.xplt
   3. Pre_operative_reverse_model.fsps
   4. Pre_operative_reverse_model.xplt
   5. Post_operative_reverse_model.fsps
   6. Post_operative_reverse_model.xplt
   7. The *.xplt files contain the results of running the corresponding *.fs2 files. If the *.xplt files were not created by running the *.fs2 files, they must be opened first before the *.fsps files to load the results.
2. Once the post-session files have been opened, play the model using the play button in the top toolbar to store and compute the exportable data.
3. After playing the model, right-click on a point probe or muscle line in the *View* tab on the left and select *Export Data*.
   1. A dialog box will open with an option to export the selected point probe/muscle line or all point probes/muscle lines.
   2. Export data for all point probes and muscle lines as two separate files.
      1. The default format for the point probe and muscle lines data is *.txt and *.csv, respectively.
         1. Note: The file format for the point probes should be changed to *.csv to work with the provided MATLAB code.
      2. Exported files should automatically be created in the ‘jobs’ folder.
4. To check your solutions, pre-solved *.csv files for the output point probe and muscle line data are provided in this repository in the folder ‘provided_solutions_FEBio’.

**Using the provided MATLAB *.m scripts and output files to calculate moment arms**

1. Required model files from this repository (all placed within the same folder directory):
   1. Verification_validation_outputs.m takes as input:
      1. Verification_validation_muscles.csv
      2. Verification_validation_probes.csv
   2. Pre_operative_reverse_outputs.m takes as input:
      1. Pre_operative_reverse_muscles.csv
      2. Pre_operative_reverse_probes.csv
   3. Post_operative_reverse_outputs.m takes as input:
      1. Post_operative_reverse_muscles.csv
      2. Post_operative_reverse_probes.csv
   4. Each analysis code comes pre-populated with the names of the exported point probe and muscle line data in the variables ‘muscle_path_file_name’ and ‘point_probe_file_name’ (lines 16 and 17, respectively), as well as the name of the output file in the ‘writecell’ command on line 258 of the script. Both of these can be altered by the user, depending on their preferred naming scheme.
2. Run the MATLAB script, and the muscle length and moment arm outputs will be computed and stored in the designated *.csv file.
   1. Alternatively, pre-solved output *.csv files can be found in ‘provided_solutions_Matlab’ for comparison. (e.g., Verification_validation_outputs.csv, Pre_operatative_reverse_outputs.csv, Post_opererative_reverse_outputs.csv

**Data provided in this repository was generated with support from the National Institute of**

**Arthritis and Musculoskeletal and Skin Diseases (NIAMS) of the National Institutes of Health**

**under award number R01 AR067196, and a Shared Instrumentation Grant S10 OD021644.**

**Development of FEBio (Finite Elements for Biomechanics and Biophysics) was supported by the National Institute of General Medical Sciences (NIGMS) of the National Institutes of Health under award number R01 GM083925.**

**Moment arm analysis code was developed with support from the National Institute of Arthritis and Musculoskeletal and Skin Diseases (NIAMS) of the National Institutes of Health under award number R01 and R56 AR067196, and a grant from the LS Peery Foundation (University of Utah Department of Orthopaedics).**
